# Supplementary material for: Integrated in vitro transcription and oligo-dT affinity chromatography enable multi-cycle reagent recycling for mRNA manufacturing
Source: Mol Ther Nucleic Acids. 2026 Sep 3;37(4):103089. doi: 10.1016/j.omtn.2026.103089 (PMC13602715; doi:10.1016/j.omtn.2026.103089)
Supplement: Supplementary file 1 [file mmc1.pdf]

OMTN, Volume *37*

Supplemental information

Integrated *in vitro* transcription and oligo-dT affinity chromatography enable multi-cycle reagent recycling for mRNA manufacturing

Adithya Nair, Jixin Qu, Manoj Pohare, Thomas C. Minshull, Caroline A. Evans, Klaudia M. Slowik, Kate A. Loveday, Zoe V.R. Ashcroft, Mabrouka Maamra, Helen E. Colley, Craig Murdoch, Mark J. Dickman, and Zoltán Kis

Template DNA Sequences

Enhanced green fluorescent protein (eGFP) sequence

AGGAAATAAGAGAGAAAAGAAGAGTAAGAAGAAATATAAGAGCCACCATGGTGAGCAAGGGCGA
GGAGCTGTTCACCGGGGTGGTGCCCATCCTGGTCGAGCTGGACGGCGACGTAAACGGCCACAA
GTTCAGCGTGTCCGGCGAGGGCGAGGGCGATGCCACCTACGGCAAGCTGACCCTGAAGTTCAT
CTGCACCACCGGCAAGCTGCCCGTGCCCTGGCCCACCCTCGTGACCACCCTGACCTACGGCGT
GCAGTGCTTCAGCCGCTACCCCGACCACATGAAGCAGCACGACTTCTTCAAGTCCGCCATGCCC
GAAGGCTACGTCCAGGAGCGCACCATCTTCTTCAAGGACGACGGCAACTACAAGACCCGCGCC
GAGGTGAAGTTCGAGGGCGACACCCTGGTGAACCGCATCGAGCTGAAGGGCATCGACTTCAAG
GAGGACGGCAACATCCTGGGGCACAAGCTGGAGTACAACTACAACAGCCACAACGTCTATATCA
TGGCCGACAAGCAGAAGAACGGCATCAAGGTGAACTTCAAGATCCGCCACAACATCGAGGACG
GCAGCGTGCAGCTCGCCGACCACTACCAGCAGAACACCCCCATCGGCGACGGCCCCGTGCTGC
TGCCCGACAACCACTACCTGAGCACCCAGTCCGCCCTGAGCAAAGACCCCAACGAGAAGCGCG
ATCACATGGTCCTGCTGGAGTTCGTGACCGCCGCCGGGATCACTCTCGGCATGGACGAGCTGT
ACAAGTAAGCGGCCGCTTAATTAAGCTGCCTTCTGCGGGGCTTGCCTTCTGGCCATGCCCTTCT
TCTCTCCCTTGCACCTGTACCTCTTGGTCTTTGAATAAAGCCTGAGTAGGAAGCGTACGAAAAAA
AAAAAAAAAAAAAAAAAAAAAAAAAAAAAAAAAAAAAAAAAA

SARS-CoV-2 Spike protein (CSP) sequence

AGAATAAACTAGTATTCTTCTGGTCCCCACAGACTCAGAGAGAACCCGCCACCATGTTCGTGTTC
CTGGTGCTGCTGCCTCTGGTGTCCAGCCAGTGTGTGAACCTGACCACCAGAACACAGCTGCCTC
CAGCCTACACCAACAGCTTTACCAGAGGCGTGTACTACCCCGACAAGGTGTTCAGATCCAGCGT
GCTGCACTCTACCCAGGACCTGTTCCTGCCTTTCTTCAGCAACGTGACCTGGTTCCACGCCATC
CACGTGTCCGGCACCAATGGCACCAAGAGATTCGACAACCCCGTGCTGCCCTTCAACGACGGG
GTGTACTTTGCCAGCACCGAGAAGTCCAACATCATCAGAGGCTGGATCTTCGGCACCACACTGG
ACAGCAAGACCCAGAGCCTGCTGATCGTGAACAACGCCACCAACGTGGTCATCAAAGTGTGCGA
GTTCCAGTTCTGCAACGACCCCTTCCTGGGCGTCTACTACCACAAGAACAACAAGAGCTGGATG
GAAAGCGAGTTCCGGGTGTACAGCAGCGCCAACAACTGCACCTTCGAGTACGTGTCCCAGCCTT
TCCTGATGGACCTGGAAGGCAAGCAGGGCAACTTCAAGAACCTGCGCGAGTTCGTGTTTAAGAA
CATCGACGGCTACTTCAAGATCTACAGCAAGCACACCCCTATCAACCTCGTGCGGGATCTGCCT
CAGGGCTTCTCTGCTCTGGAACCCCTGGTGGATCTGCCCATCGGCATCAACATCACCCGGTTTC
AGACACTGCTGGCCCTGCACAGAAGCTACCTGACACCTGGCGATAGCAGCAGCGGATGGACAG
CTGGTGCCGCCGCTTACTATGTGGGCTACCTGCAGCCTAGAACCTTCCTGCTGAAGTACAACGA
GAACGGCACCATCACCGACGCCGTGGATTGTGCTCTGGATCCTCTGAGCGAGACAAAGTGCAC
CCTGAAGTCCTTCACCGTGGAAAAGGGCATCTACCAGACCAGCAACTTCCGGGTGCAGCCCACC
GAATCCATCGTGCGGTTCCCCAATATCACCAATCTGTGCCCCTTCGGCGAGGTGTTCAATGCCA
CCAGATTCGCCTCTGTGTACGCCTGGAACCGGAAGCGGATCAGCAATTGCGTGGCCGACTACTC
CGTGCTGTACAACTCCGCCAGCTTCAGCACCTTCAAGTGCTACGGCGTGTCCCCTACCAAGCTG
AACGACCTGTGCTTCACAAACGTGTACGCCGACAGCTTCGTGATCCGGGGAGATGAAGTGCGG
CAGATTGCCCCTGGACAGACAGGCAAGATCGCCGACTACAACTACAAGCTGCCCGACGACTTCA
CCGGCTGTGTGATTGCCTGGAACAGCAACAACCTGGACTCCAAAGTCGGCGGCAACTACAATTA
CCTGTACCGGCTGTTCCGGAAGTCCAATCTGAAGCCCTTCGAGCGGGACATCTCCACCGAGATC
TATCAGGCCGGCAGCACCCCTTGTAACGGCGTGGAAGGCTTCAACTGCTACTTCCCACTGCAGT
CCTACGGCTTTCAGCCCACAAATGGCGTGGGCTATCAGCCCTACAGAGTGGTGGTGCTGAGCTT
CGAACTGCTGCATGCCCCTGCCACAGTGTGCGGCCCTAAGAAAAGCACCAATCTCGTGAAGAAC
AAATGCGTGAACTTCAACTTCAACGGCCTGACCGGCACCGGCGTGCTGACAGAGAGCAACAAGA
AGTTCCTGCCATTCCAGCAGTTTGGCCGGGATATCGCCGATACCACAGACGCCGTTAGAGATCC
CCAGACACTGGAAATCCTGGACATCACCCCTTGCAGCTTCGGCGGAGTGTCTGTGATCACCCCT
GGCACCAACACCAGCAATCAGGTGGCAGTGCTGTACCAGGACGTGAACTGTACCGAAGTGCCC

GTGGCCATTCACGCCGATCAGCTGACACCTACATGGCGGGTGTACTCCACCGGCAGCAATGTGT
TTCAGACCAGAGCCGGCTGTCTGATCGGAGCCGAGCACGTGAACAATAGCTACGAGTGCGACAT
CCCCATCGGCGCTGGAATCTGCGCCAGCTACCAGACACAGACAAACAGCCCTCGGAGAGCCAG
AAGCGTGGCCAGCCAGAGCATCATTGCCTACACAATGTCTCTGGGCGCCGAGAACAGCGTGGC
CTACTCCAACAACTCTATCGCTATCCCCACCAACTTCACCATCAGCGTGACCACAGAGATCCTGC
CTGTGTCCATGACCAAGACCAGCGTGGACTGCACCATGTACATCTGCGGCGATTCCACCGAGTG
CTCCAACCTGCTGCTGCAGTACGGCAGCTTCTGCACCCAGCTGAATAGAGCCCTGACAGGGATC
GCCGTGGAACAGGACAAGAACACCCAAGAGGTGTTCGCCCAAGTGAAGCAGATCTACAAGACC
CCTCCTATCAAGGACTTCGGCGGCTTCAATTTCAGCCAGATTCTGCCCGATCCTAGCAAGCCCA
GCAAGCGGAGCTTCATCGAGGACCTGCTGTTCAACAAAGTGACACTGGCCGACGCCGGCTTCAT
CAAGCAGTATGGCGATTGTCTGGGCGACATTGCCGCCAGGGATCTGATTTGCGCCCAGAAGTTT
AACGGACTGACAGTGCTGCCTCCTCTGCTGACCGATGAGATGATCGCCCAGTACACATCTGCCC
TGCTGGCCGGCACAATCACAAGCGGCTGGACATTTGGAGCAGGCGCCGCTCTGCAGATCCCCT
TTGCTATGCAGATGGCCTACCGGTTCAACGGCATCGGAGTGACCCAGAATGTGCTGTACGAGAA
CCAGAAGCTGATCGCCAACCAGTTCAACAGCGCCATCGGCAAGATCCAGGACAGCCTGAGCAG
CACAGCAAGCGCCCTGGGAAAGCTGCAGGACGTGGTCAACCAGAATGCCCAGGCACTGAACAC
CCTGGTCAAGCAGCTGTCCTCCAACTTCGGCGCCATCAGCTCTGTGCTGAACGATATCCTGAGC
AGACTGGACCCTCCTGAGGCCGAGGTGCAGATCGACAGACTGATCACAGGCAGACTGCAGAGC
CTCCAGACATACGTGACCCAGCAGCTGATCAGAGCCGCCGAGATTAGAGCCTCTGCCAATCTGG
CCGCCACCAAGATGTCTGAGTGTGTGCTGGGCCAGAGCAAGAGAGTGGACTTTTGCGGCAAGG
GCTACCACCTGATGAGCTTCCCTCAGTCTGCCCCTCACGGCGTGGTGTTTCTGCACGTGACATA
TGTGCCCGCTCAAGAGAAGAATTTCACCACCGCTCCAGCCATCTGCCACGACGGCAAAGCCCAC
TTTCCTAGAGAAGGCGTGTTCGTGTCCAACGGCACCCATTGGTTCGTGACACAGCGGAACTTCT
ACGAGCCCCAGATCATCACCACCGACAACACCTTCGTGTCTGGCAACTGCGACGTCGTGATCGG
CATTGTGAACAATACCGTGTACGACCCTCTGCAGCCCGAGCTGGACAGCTTCAAAGAGGAACTG
GACAAGTACTTTAAGAACCACACAAGCCCCGACGTGGACCTGGGCGATATCAGCGGAATCAATG
CCAGCGTCGTGAACATCCAGAAAGAGATCGACCGGCTGAACGAGGTGGCCAAGAATCTGAACG
AGAGCCTGATCGACCTGCAAGAACTGGGGAAGTACGAGCAGTACATCAAGTGGCCCTGGTACAT
CTGGCTGGGCTTTATCGCCGGACTGATTGCCATCGTGATGGTCACAATCATGCTGTGTTGCATG
ACCAGCTGCTGTAGCTGCCTGAAGGGCTGTTGTAGCTGTGGCAGCTGCTGCAAGTTCGACGAG
GACGATTCTGAGCCCGTGCTGAAGGGCGTGAAACTGCACTACACATGATGACTCGAGCTGGTAC
TGCATGCACGCAATGCTAGCTGCCCCTTTCCCGTCCTGGGTACCCCGAGTCTCCCCCGACCTCG
GGTCCCAGGTATGCTCCCACCTCCACCTGCCCCACTCACCACCTCTGCTAGTTCCAGACACCTC
CCAAGCACGCAGCAATGCAGCTCAAAACGCTTAGCCTAGCCACACCCCCACGGGAAACAGCAGT
GATTAACCTTTAGCAATAAACGAAAGTTTAACTAAGCTATACTAACCCCAGGGTTGGTCAATTTCG
TGCCAGCCACACCCTGGAGCTAGCAAAAAAAAAAAAAAAAAAAAAAAAAAAAAAAGCATATGACTA
AAAAAAAAAAAAAAAAAAAAAAAAAAAAAAAAAAAAAAAAAAAAAAAAAAAAAAAAAAAAAAAAAA
AA

Table S1. Initial reaction compositions for standard IVT and cycle 1 of the IVT-oligo-dT workflows. Stock concentrations are listed alongside the nominal starting concentrations used in each condition.

Component	Stock concentration	Standard IVT	IVT-Oligo-dT
Tris-HCl	1000 mM	40 mM	40 mM
DTT	1000 mM	10 mM	10 mM
Spermidine	6400 mM	2 mM	2 mM
Triton X-100	100% (v/v)	0.01% (v/v)	0.01% (v/v)
[a] $NaCl/CH_3COOK$	5000/1000 mM	-	100 mM
GuHCl	8000 mM	-	50 mM
$MgCl_2$	1000 mM	42 mM	16 mM
NTP (total)	100 mM	40 mM	10 mM
CleanCap AG	100 mM	10 mM	2.5 mM
T7 RNAP	0.010 mM	0.001 mM	0.00075 mM
Template DNA	0.00050 mM	0.000025 mM	0.000025 mM
Inorganic pyrophosphatase	0.0618 mM	0.0030 mM	0.0030 mM
RNase inhibitor	0.0075 mM	0.00019 mM	0.00019 mM
RNase-free water	-	-	-

[a] $NaCl/CH_3COOK$ indicates that either NaCl or CH_3COOK was used at 100 mM. Unless otherwise stated, all other component concentrations in the IVT-oligo-dT condition are identical in both buffer systems.

Table S2. Fixed supplementation before each recycled IVT cycle (cycles 2–5).

Component	Supplemented amount per cycle
$MgCl_2$	80 µmol/cycle
NTP (total)	80 µmol/cycle (20 µmol of each NTP)
Template DNA	200 µg/cycle
All other components	-

Note: Supplementation amounts were fixed charges added before each of cycles 2-5. The 80 µmol $MgCl_2$ charge corresponds to a nominal 10 mM increment in an 8 mL reaction. Residual Mg^{2+} and NTPs from the preceding cycle were not quantified or subtracted; therefore, these values do not represent actual cycle-start concentrations. A dash indicates no between-cycle supplementation.

Table S3. Buffer compositions used for standard oligo-dT and integrated IVT-oligo-dT purification methods.

	**Component**	**Standard oligo-dT**	**IVT-Oligo-dT NaCl buffer**	**IVT-Oligo-dT CH_3COOK buffer**
Equilibration	NaCl	250 mM	100 mM	-
	Phosphate buffer	50 mM	-	-
	EDTA	2 mM	-	-
	Gu-HCl	-	50 mM	50 mM
	Tris-HCl (pH 7.0)	-	40 mM	40 mM
	CH_3COOK	-	-	100 mM
Load	Same as equilibration			
Wash	Same as equilibration			
Elution	Nuclease-free water			
CIP	NaOH		100 mM	

Table S4. Cap analog concentration in flowthrough and residual NTP in elution.

Cycle	Cap analog in flowthrough (NaCl buffer, % relative to cycle 1)	Cap analog in flowthrough (CH_3COOK buffer, % relative to cycle 1)	Residual rNTP in elution (NaCl buffer, %)	Residual rNTP in elution (CH_3COOK buffer, %)
1	100.0	100.0	Not detected	0.4
2	75.9	70.7	Not detected	0.8
3	75.4	60.3	Not detected	0.5
4	54.4	50.9	Not detected	0.3
5	36.9	39.4	Not detected	0.2

Table S5. Source data supporting the main figures. The underlying numerical values used for the quantitative analyses are provided as a separate Excel workbook.

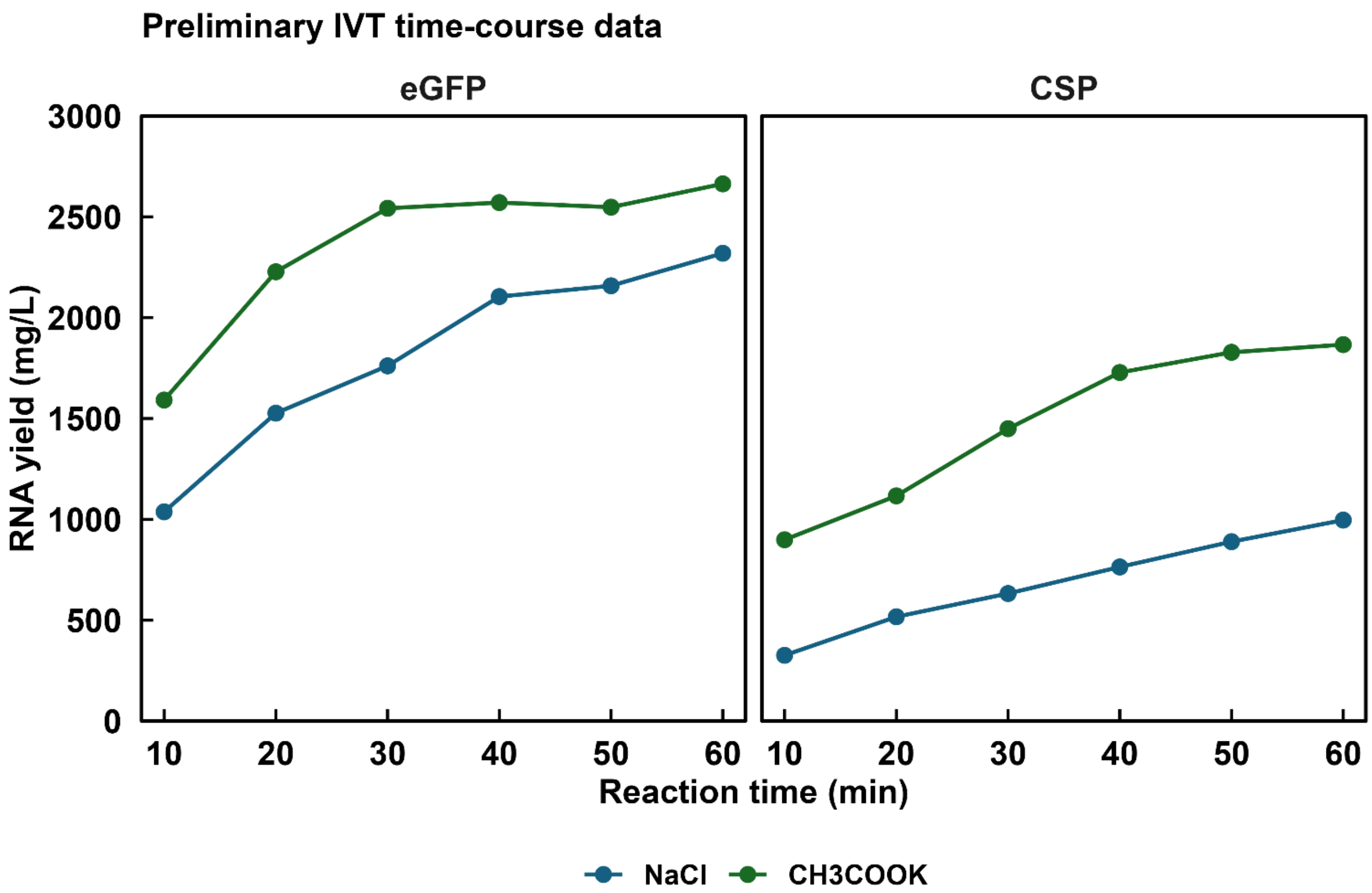

Figure S1. IVT time-course screening under shared-buffer conditions. RNA yield was measured over time for eGFP and CSP transcripts in NaCl- and CH_3COOK-based shared buffers containing 50 mM Gu-HCl. The data were used as preliminary process-development screening to support the buffer-dependent incubation-time rationale in the integrated IVT-oligo-dT recycling workflow. CH_3COOK showed faster apparent RNA accumulation than NaCl under the tested conditions. Data are preliminary screening measurements and were not used for statistical comparison.

A

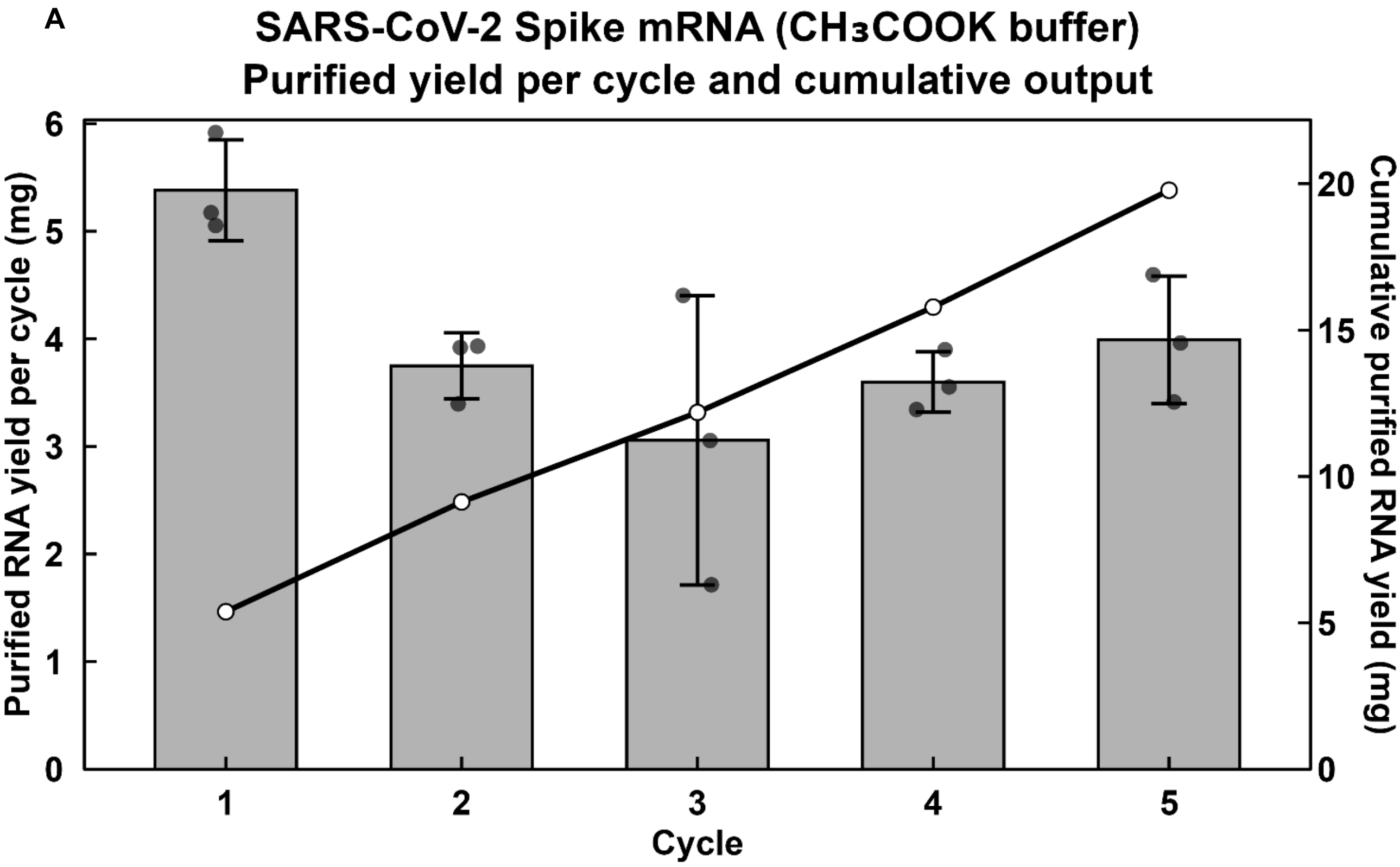

SARS-CoV-2 Spike mRNA (CH₃COOK buffer)
Purified yield per cycle and cumulative output
Purified RNA yield per cycle (mg)
Cumulative purified RNA yield (mg)
Cycle
0
1
2
3
4
5
6
10
15
20

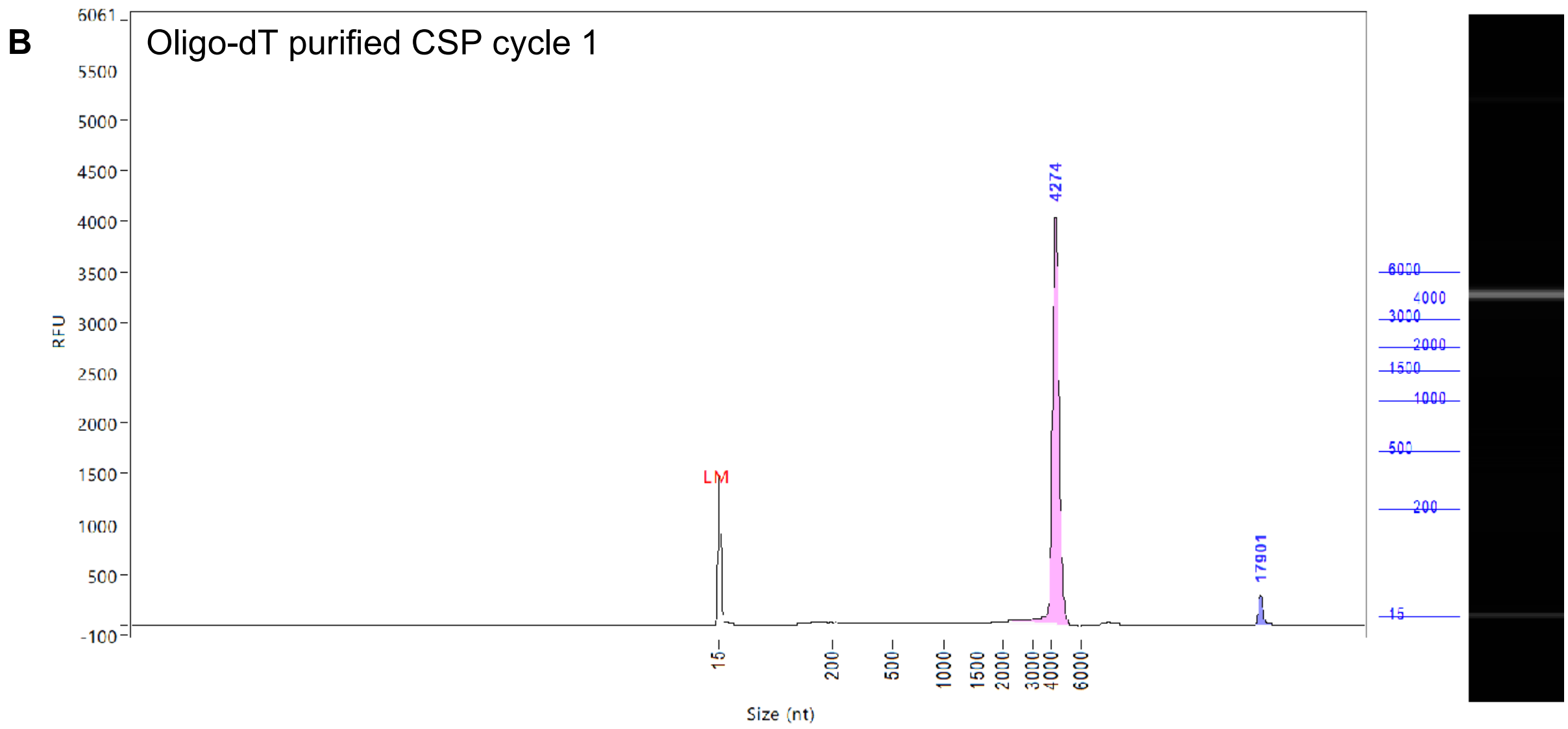

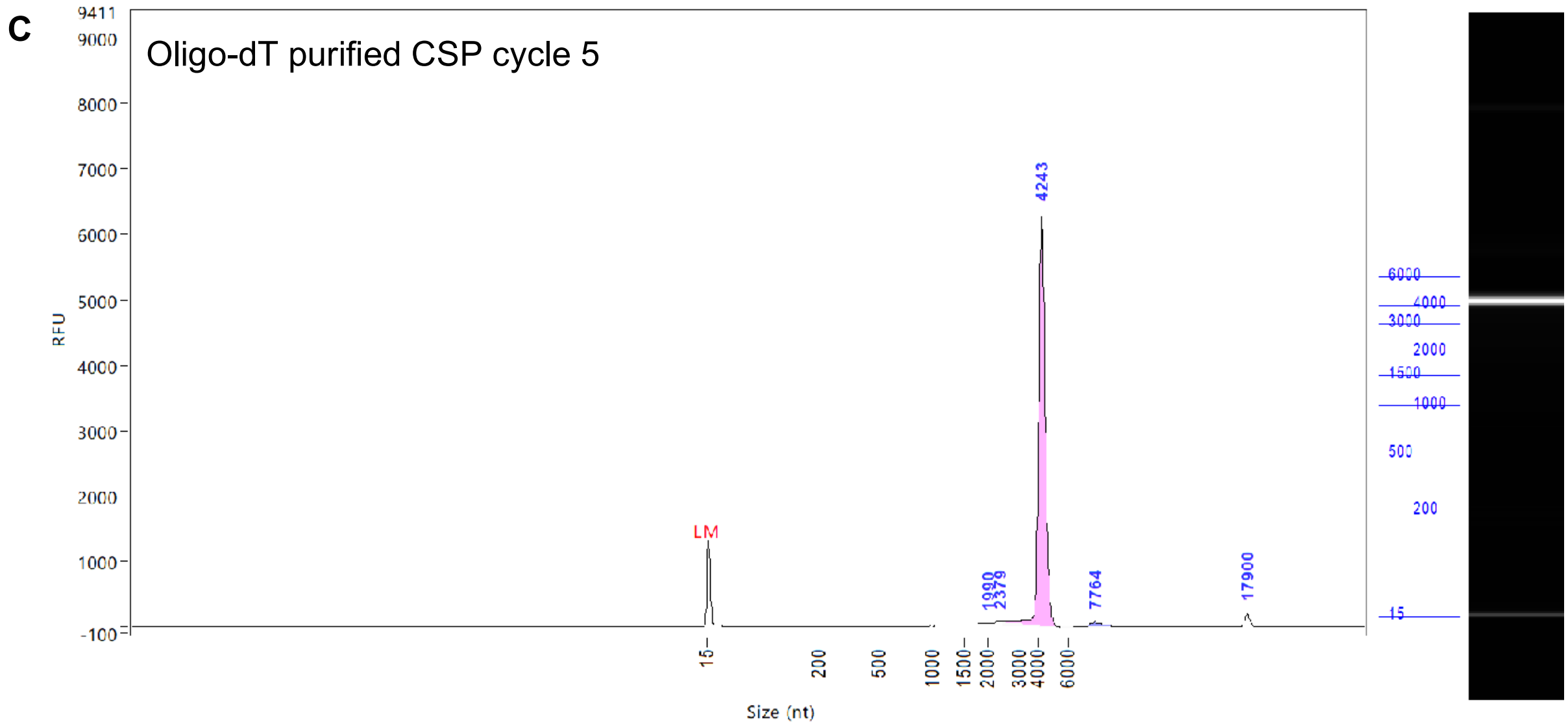

Figure S2. Feasibility of integrated IVT-oligo-dT recycling with a longer CSP transcript**. A,** Purified RNA yield per cycle and cumulative purified RNA output for SARS-CoV-2 Spike protein (CSP) mRNA produced using the CH_3COOK-based recycling workflow. Bars show purified RNA yield obtained in each cycle, with individual replicate values overlaid as dots; error bars indicate standard deviation. The black line with open circles represents cumulative purified RNA yield across successive cycles, referenced to the right y-axis. **B,C,** Representative electropherograms of purified CSP mRNA recovered after oligo-dT chromatography from cycle 1 and cycle 5, respectively. The comparable electropherogram profiles are consistent with preserved purified-product integrity across the recycling process. Crude CSP electropherograms were not available; therefore, these data were not used for crude-to-purified integrity comparison.

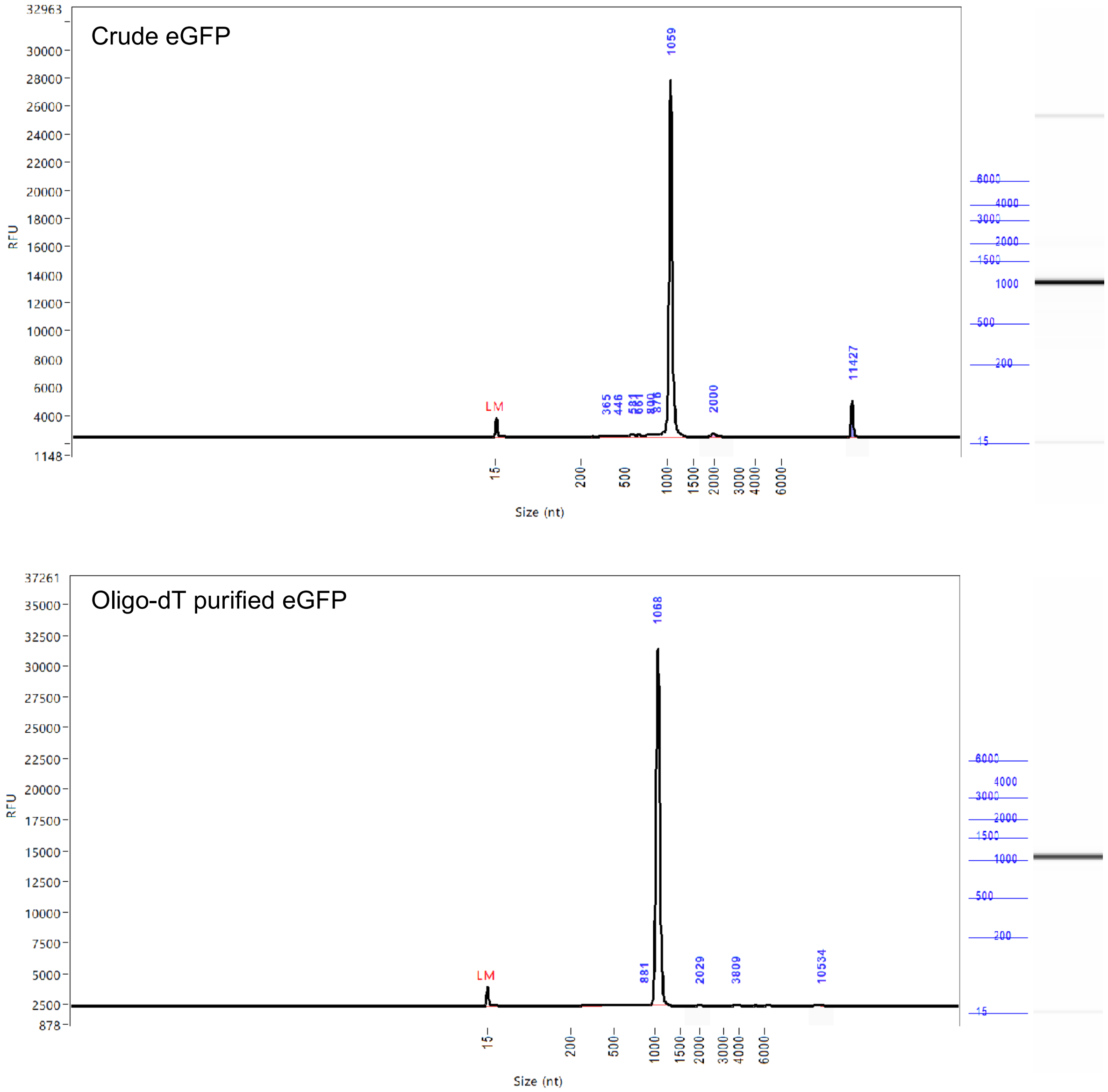

Figure S3. Representative electropherograms of crude and purified eGFP mRNA. Representative electropherograms are shown for crude eGFP mRNA after IVT and purified eGFP mRNA after oligo-dT chromatography.

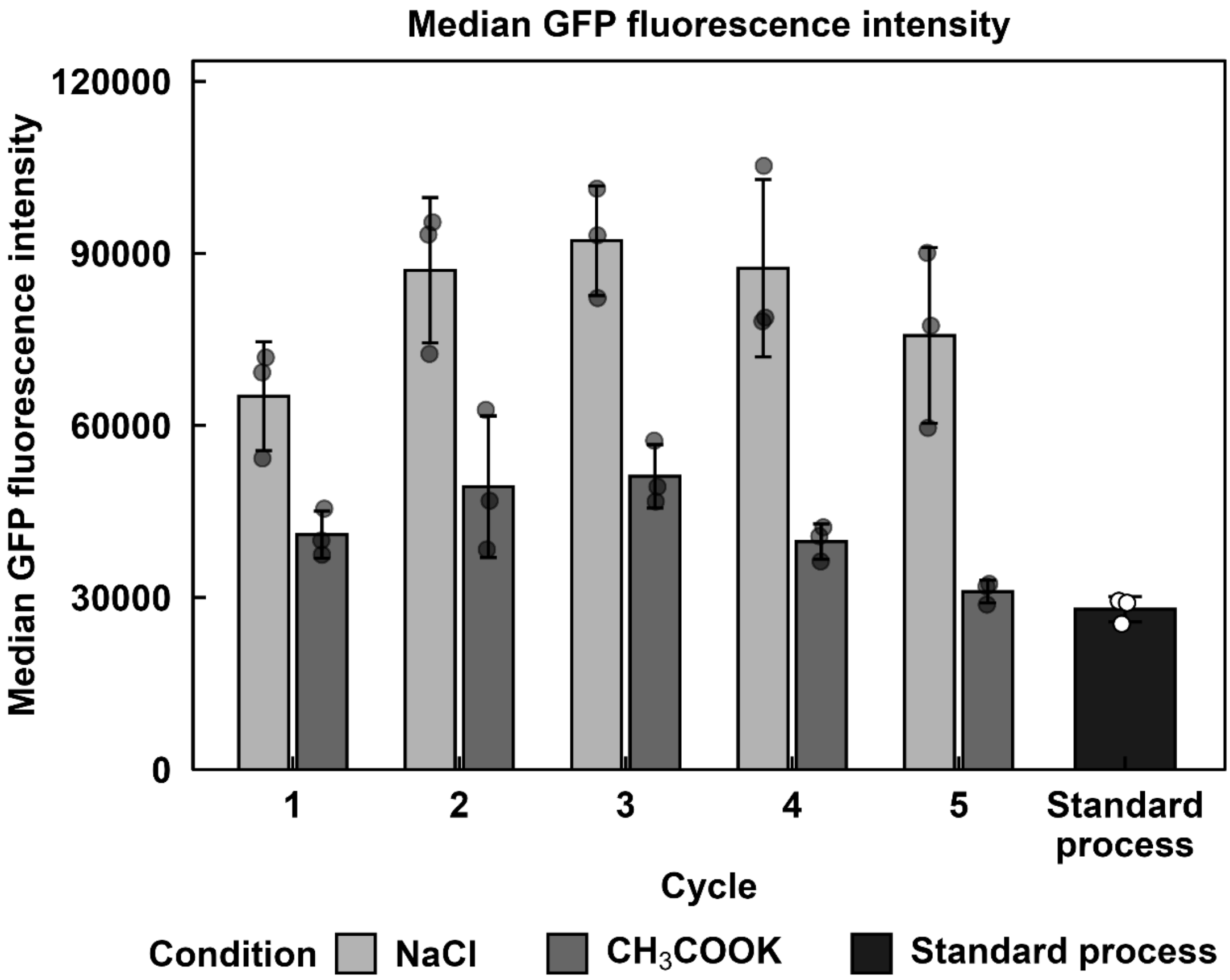

Figure S4. Median GFP fluorescence intensity following transfection with recycled mRNA**.** Median GFP fluorescence intensity (MFI) was measured in THP-1 cells transfected with eGFP mRNA generated using the standard batch IVT process or the integrated IVT-oligo-dT recycling workflow with NaCl or CH_3COOK buffers. Bars show mean values from three independent transfection experiments, with individual replicate values overlaid as points; error bars indicate SD. The data provide a complementary protein-expression readout to the percentage of eGFP-positive cells shown in **Figure 6A**.
