# Supplementary material for: Integrated in vitro transcription and oligo-dT affinity chromatography enable multi-cycle reagent recycling for mRNA manufacturing
Source: Mol Ther Nucleic Acids. 2026 Sep 3;37(4):103089. doi: 10.1016/j.omtn.2026.103089 (PMC13602715; doi:10.1016/j.omtn.2026.103089)
Supplement: Supplementary file 2 [file mmc3.pdf]

Molecular Therapy
Nucleic Acids
Original Article

Integrated *in vitro* transcription and oligo-dT affinity chromatography enable multi-cycle reagent recycling for mRNA manufacturing

Adithya Nair,[1,4] Jixin Qu,[1,4] Manoj Pohare,[1] Thomas C. Minshull,[1] Caroline A. Evans,[1] Klaudia M. Slowik,[3] Kate A. Loveday,[1] Zoe V.R. Ashcroft,[1] Mabrouka Maamra,[1] Helen E. Colley,[3] Craig Murdoch,[3] Mark J. Dickman,[1] and Zoltán Kis[1,2]

[1]School of Chemical, Materials and Biological Engineering, University of Sheffield, Mappin Street, Sheffield S1 3JD, UK; [2]Department of Chemical Engineering, Imperial College London, South Kensington Campus, London SW7 2AZ, UK; [3]School of Clinical Dentistry, University of Sheffield, 19 Claremont Crescent, Sheffield S10 2TA, UK

A platform process underpins the manufacturing of RNA-based vaccines and therapeutics. However, it remains constrained by high operational expenditures from costly reagents, inefficient raw-material utilization, and extensive purification. We report an integrated sequential-batch *in vitro* transcription (IVT)-oligo-dT chromatography process that links RNA synthesis and purification via a shared buffer, enabling reagent recycling. Across five cycles, 5′ cap analog utilization improved 3.72× and calculated raw-material cost efficiency improved 2.21× for the NaCl workflow at the demonstrated 8 mL scale, while stable RNA production was maintained across recycling cycles. Product quality was assessed across seven critical quality attributes (CQAs), including RNA integrity, 5′ capping, poly(A)-tail heterogeneity, sequence identity, residual nucleotides, dsRNA content, and cell-based functional activity, measured by protein expression and cytokine responses. Purified recycling-process RNA contained ≈ 70%–77% less double-stranded RNA (dsRNA) than purified standard-process RNA, purified RNA integrity exceeded 90%, and five-cycle mean 5′ capping exceeded 80% with NaCl; 3′ polyadenylate (poly(A)) tail length and heterogeneity remained stable across recycling cycles. THP-1 cell assays showed no progressive cycle-dependent loss of protein expression or increase in cytokine secretion across recycling cycles. This integrated framework advances cost- and resource-efficient mRNA production while identifying candidate control points for extended reagent recycling.

INTRODUCTION

RNA-based medicines are rapidly emerging as an important class of therapeutics, including vaccines and therapies for a wide range of diseases. The clinical success of messenger RNA (mRNA)-based COVID-19 vaccines reinvigorated interest in RNA therapeutics and accelerated exploration of their potential applications. Beyond prophylactic vaccines, RNA therapeutics are poised to enable therapeutic protein replacement, immune modulation, and regenerative medicine. In these latter contexts, RNA is increasingly used for cell engineering applications, including immune-cell programming for adoptive cell therapies (e.g., CAR-T).[1–3] Additionally, RNA plays a central role in gene-editing strategies, where guide RNAs direct CRISPR-associated nucleases to genomic targets. These diverse applications, spanning mRNA, self-amplifying RNA (saRNA), circular RNA (circRNA), and small guide RNAs (sgRNA), are driving unprecedented demand for scalable, high-quality, and cost-efficient RNA manufacturing processes.[4–6] Although the process developed here is most directly applicable to poly(A)-tailed mRNA, this broader therapeutic landscape underscores the need for manufacturing strategies that improve raw-material utilization, process efficiency, and product quality.

RNA modalities are attractive because their manufacturing processes are relatively simple and highly adaptable. The same production platform can be used to manufacture many different RNA sequences, which supports rapid deployment and scale-up across applications.[7–9] Manufacturing is broadly divided into upstream drug substance (DS) synthesis and downstream purification and formulation into the final drug product (DP).[10,11] Synthesis occurs via *in vitro* transcription (IVT), a cell-free enzymatic process that employs a DNA template encoding the gene of interest (GOI), a DNA-dependent RNA polymerase (typically bacteriophage-derived, such as T7, T3, or SP6), nucleotide triphosphates (NTPs), cofactors, and other reaction modulators.[12–14] Standardized components and reaction simplicity enable a sequence-agnostic platform for diverse RNA DS production.

The resulting mRNA contains a 5′ cap and 3′ poly(A) tail, both of which can be incorporated co-transcriptionally during IVT or

Received 20 May 2026; accepted 2 September 2026;
https://doi.org/10.1016/j.omtn.2026.103089.

[4]These authors contributed equally

Correspondence: Adithya Nair, School of Chemical, Materials and Biological Engineering, University of Sheffield, Mappin Street, Sheffield S1 3JD, UK.
E-mail: adithya.nair@sheffield.ac.uk
Correspondence: Zoltán Kis, School of Chemical, Materials and Biological Engineering, University of Sheffield, Mappin Street, Sheffield S1 3JD, UK.
E-mail: z.kis@sheffield.ac.uk

Published by Elsevier Inc. on behalf of The American Society of Gene and Cell Therapy.
This is an open access article under the CC BY license (http://creativecommons.org/licenses/by/4.0/).

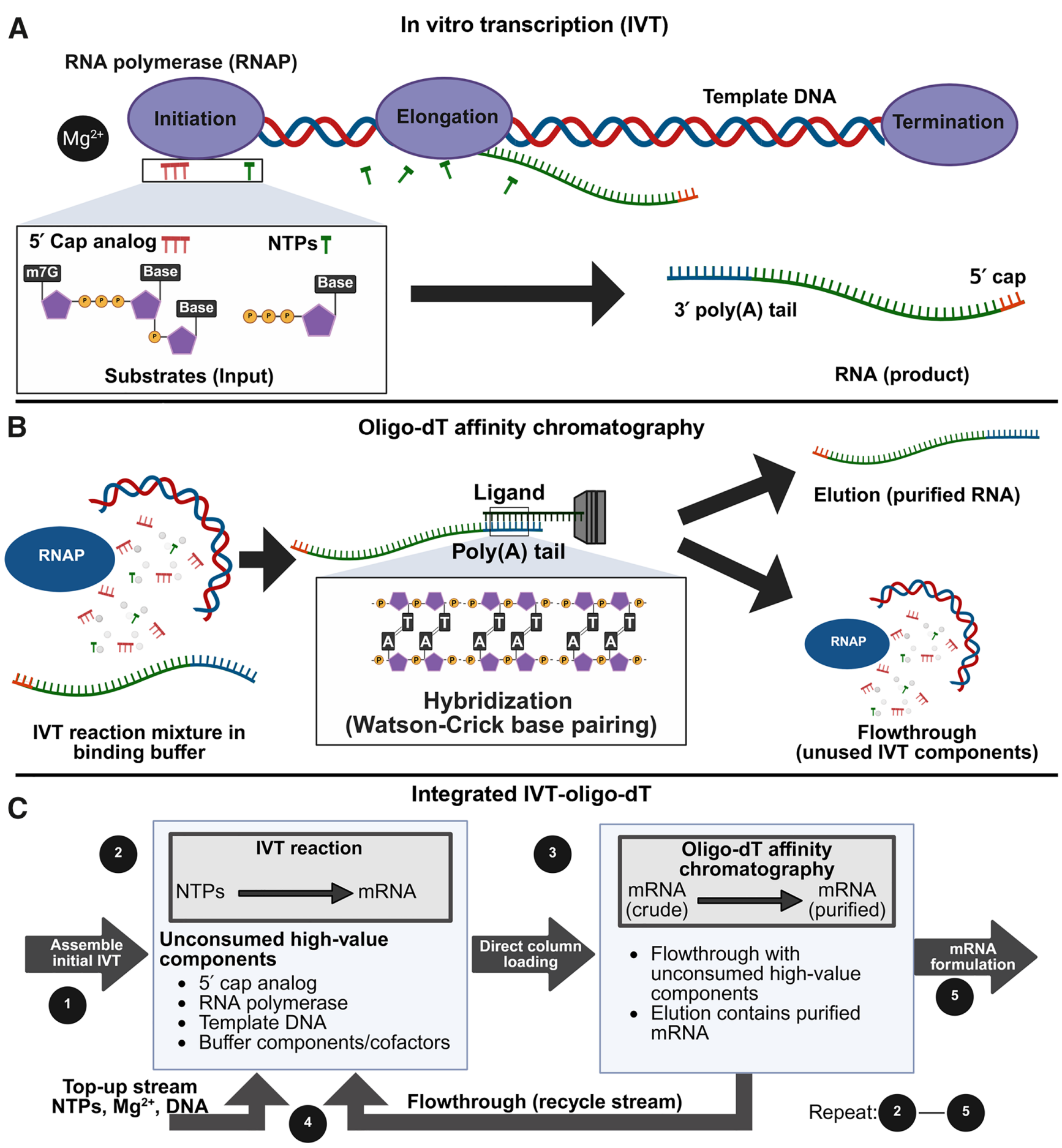

Figure 1. Integrated sequential-batch IVT-oligo-dT recycling workflow

(A) IVT in a shared buffer containing the DNA template, NTPs, 5′ cap analog, T7 RNA polymerase, and magnesium ions. (B) Direct loading of the IVT mixture onto an oligo-dT column. Poly(A)-tailed RNA is captured and eluted, while unbound IVT components pass into the flowthrough. (C) Sequential-batch recycling in which the pooled recycle stream receives predefined NTP and $MgCl_2$ additions and a separate template-DNA top-up before reuse in the subsequent IVT cycle. Created in BioRender, https://BioRender.com/8r08g4y.

enzymatically added post-transcriptionally. The co-transcriptional route is often preferred because it simplifies upstream operations.[7] In this approach, the poly(A) tail is encoded in the DNA template, and the 5′ cap is introduced using synthetic GAG-type trinucleotide cap analogs such as CleanCap AG (TriLink Biotechnologies). Pfizer-BioNTech's COVID-19 vaccine (BNT162b2) employed the same co-transcriptional capping strategy with CleanCap AG, underscoring the clinical maturity of this method.[15–17] Although only a single cap analog is required per RNA molecule, efficient capping requires analog concentrations comparable with those of NTPs. In representative techno-economic analyses, raw materials constitute ≈74% of total operating expenditure (OpEx),[15,18,19] with CleanCap AG accounting for ≈36% of that spend. In conventional batch IVT manufacturing, most of the unreacted 5′ cap analog (>99%), together with potentially reusable enzymes and DNA templates, is discarded after each batch. This loss of high-value reagents is the inefficiency that motivates strategies for recovery and reuse.

Once the RNA DS is synthesized, it undergoes purification to remove process- and product-related impurities. Common strategies include tangential flow filtration (TFF)[20–22] and chromatographic techniques, including affinity and ion-pair reverse-phase chromatography.[23–26] Oligodeoxythymidinic acid acid (oligo-dT) affinity chromatography exploits base pairing between the poly(A) tail and the oligo-dT ligand, operating under elevated-ionic-strength loading conditions (0.5–1 M) and low-salt elution (a suitable buffer or RNase-free water).[27–30] Although current manufacturing is technically effective, it remains economically and resource inefficient because high-value unconsumed IVT reagents are largely discarded after each batch. Integrating RNA synthesis with affinity purification can combine product capture with recovery of unused reagents, reducing waste and enabling more sustainable manufacturing.

Here, we demonstrate an integrated IVT-oligo-dT affinity chromatography process that couples RNA synthesis and purification through a shared buffer system. This enables crude IVT mixtures to be loaded directly onto the purification column without intermediate buffer exchange or cleanup. In this context, "integrated" refers to chemical and operational coupling through shared-buffer compatibility, direct crude-IVT loading, and reuse of the oligo-dT flowthrough as the reaction matrix for the subsequent IVT cycle. The demonstrated workflow remains batchwise in execution and is distinct from simultaneous reaction-separation coupling or fully continuous manufacturing. Importantly, unused IVT reagents that pass through the column are collected and reused in subsequent transcription cycles. To our knowledge, no prior study has demonstrated the combined direct loading of crude IVT onto an oligo-dT affinity column and reuse of the chromatography flowthrough as the reaction medium for subsequent IVT cycles. Figure 1 illustrates the concept of the integrated IVT-oligo-dT recycling process. In the first step, RNA is synthesized by IVT using a DNA template, nucleotide triphosphates, and a 5′ cap analog. The resulting reaction mixture is then directly loaded onto an oligo-dT affinity column using a buffer compatible with both transcription and affinity binding. During purification, poly(A)-tailed RNA binds to the oligo-dT stationary phase, while unused IVT components, including the 5′ cap analog, RNA polymerase, DNA template, and small-molecule cofactors, pass through the

column. This flowthrough is collected, supplemented with depleted substrates such as NTPs and Mg^{2+}, and reused as the reaction matrix for subsequent IVT cycles. The integrated process therefore links direct crude-IVT loading, oligo-dT capture, and flowthrough reuse without dilution or intermediate buffer exchange. The process development considered critical material attributes (CMAs) and critical process parameters (CPPs) to assess their impact on manufacturing key performance indicators (KPIs) and product critical quality attributes (CQAs). KPIs are associated with manufacturing productivity and cost, whereas CQAs are properties of the RNA DS that affect patient safety, product efficacy, and process comparability.

In addition to quantifying process performance, raw-material utilization, and cost reduction, we assessed whether integration preserved mRNA DS quality across repeated recycling cycles. Seven CQAs were evaluated: RNA integrity, 5′ capping efficiency, RNA sequence identity, residual nucleotides, poly(A)-tail length and heterogeneity, double-stranded RNA (dsRNA) content, and cell-based functional activity measured by protein expression and cytokine responses. The integrated process reduced raw-material costs while maintaining stable cycle-wise RNA production and a broad mRNA quality profile, establishing a foundation for economically and resource-efficient RNA manufacturing.

RESULTS

To systematically evaluate the performance of the integrated IVT-oligo-dT recycling process, the results are presented in four stages. First, we assess process performance, including RNA yield, productivity, and operational stability across recycling cycles. Second, we quantify the economic impact of reagent recycling by analyzing raw-material utilization and production costs. Third, we evaluate whether the integrated workflow preserves mRNA CQAs, including RNA integrity, poly(A)-tail heterogeneity, sequence identity, 5′ capping efficiency, and dsRNA content. Finally, we assess cell-based functional activity, including protein expression and cytokine responses to the resulting mRNA. This structure allows the process performance, economic benefits, and product quality of the integrated workflow to be evaluated in a logical progression.

Process performance: Impact of IVT-oligo-dT integration on RNA yield and productivity

Integrating IVT and oligo-dT capture requires a shared buffer that supports transcription while enabling on-column poly(A)-oligo-dT hybridization. We evaluated sodium chloride (NaCl) and potassium acetate (CH_3COOK)-based buffers over five sequential IVT-oligo-dT cycles. Cycle 1 was initiated with equimolar NTPs (2.5 mM each; 10 mM total) and CleanCap AG (2.5 mM; 20 μmol in 8 mL). Before cycles 2–5, the pooled flowthrough received fixed additions of 80 μmol total NTPs (20 μmol each) and 80 μmol $MgCl_2$, together with a separate 200 μg template-DNA top-up. Neither CleanCap AG nor T7 RNA polymerase was replenished; subsequent cycles relied on these components remaining in the recycled flowthrough. An 8 mL standard high-yield IVT followed by oligo-dT after buffer exchange served as the benchmark. The NaCl- and CH_3COOK-based workflows used different IVT incubation times because preliminary development indicated buffer-dependent apparent RNA-accumulation kinetics and informed buffer-specific operating points. The selected durations balanced RNA yield, cycle time, and compatibility with direct oligo-dT loading; they were not matched kinetic endpoints, and equivalent reaction completion or plateau conditions were not established. The objective was to evaluate the configured recycling workflows rather than maximize single-cycle yield. Preliminary screening showed faster apparent RNA accumulation with CH_3COOK than with NaCl under the tested shared-buffer conditions (Figure S1).

RNA yields for each cycle are shown in Figure 2A. Individual IVT-oligo-dT cycles took 2.17 h in NaCl (1.50 h IVT + 0.67 h oligo-dT) and 1.08 h in CH_3COOK (0.41 h IVT + 0.67 h oligo-dT), totaling 10.85 h and 5.40 h over five cycles, respectively. By comparison, the standard 8 mL process yielded 84.3 ± 0.4 mg purified RNA in 4.0 h (2.0 h IVT + 2.0 h oligo-dT), corresponding to a production rate of 21.1 mg h^{-1} and productivity of 2.63 mg mL^{-1} h^{-1}. The longer oligo-dT step in the standard process reflects adjustment of the crude IVT to the higher-salt loading condition it requires (Table S3), which the integrated workflow avoids by loading directly. Cumulative purified RNA yields were 78.5 ± 4.9 mg (NaCl) and 68.6 ± 4.4 mg (CH_3COOK), compared with 84.3 ± 0.4 mg for the standard process. Recovery after oligo-dT averaged 87.3% ± 2.0% (NaCl) and 85.0% ± 7.1% (CH_3COOK) versus 95% for the standard process (Figure 2B).

Between buffers, the cumulative difference favored NaCl but was not statistically significant (Δ = NaCl − CH_3COOK = +9.82 mg, 95% CI [−0.84 to 20.49]; two-sided Welch's t test, $p = 0.063$). Relative to the standard process, cumulative yield was 5.81 mg lower with NaCl (95% CI [−6.36 to 17.97]) and 15.63 mg lower with CH_3COOK (95% CI [4.76–26.49]; two-sided Welch's t tests, $n = 3$ independent experiments). No statistically detectable cycle effect on purified RNA yield was found for either buffer (Kruskal-Wallis: CH_3COOK, $H(4) = 7.37$, $p = 0.118$; NaCl, $H(4) = 4.43$, $p = 0.351$). Variation across the five cycle means was low (<10% coefficient of variation), and no monotonic decline was evident (Figure 2A). This sustained per-cycle output demonstrates retained net process functionality across five cycles, although active T7 RNA polymerase recovery was not directly quantified. The CH_3COOK workflow also produced purified RNA from the longer CSP transcript in each recycling cycle (Figure S2A). Taken together, these results identify shared-buffer salt identity as a candidate CMA linked to the selected operating point and yield-rate trade-off. Under the configured conditions, NaCl produced the higher mean cumulative yield, whereas CH_3COOK used a shorter cycle time and achieved a higher calculated production rate. Because the workflows were not compared at matched kinetic endpoints, these differences should not be interpreted as intrinsic salt effects at equivalent reaction completion.

In summary, these results demonstrate that the integrated IVT and oligo-dT workflow maintains sustained RNA production across

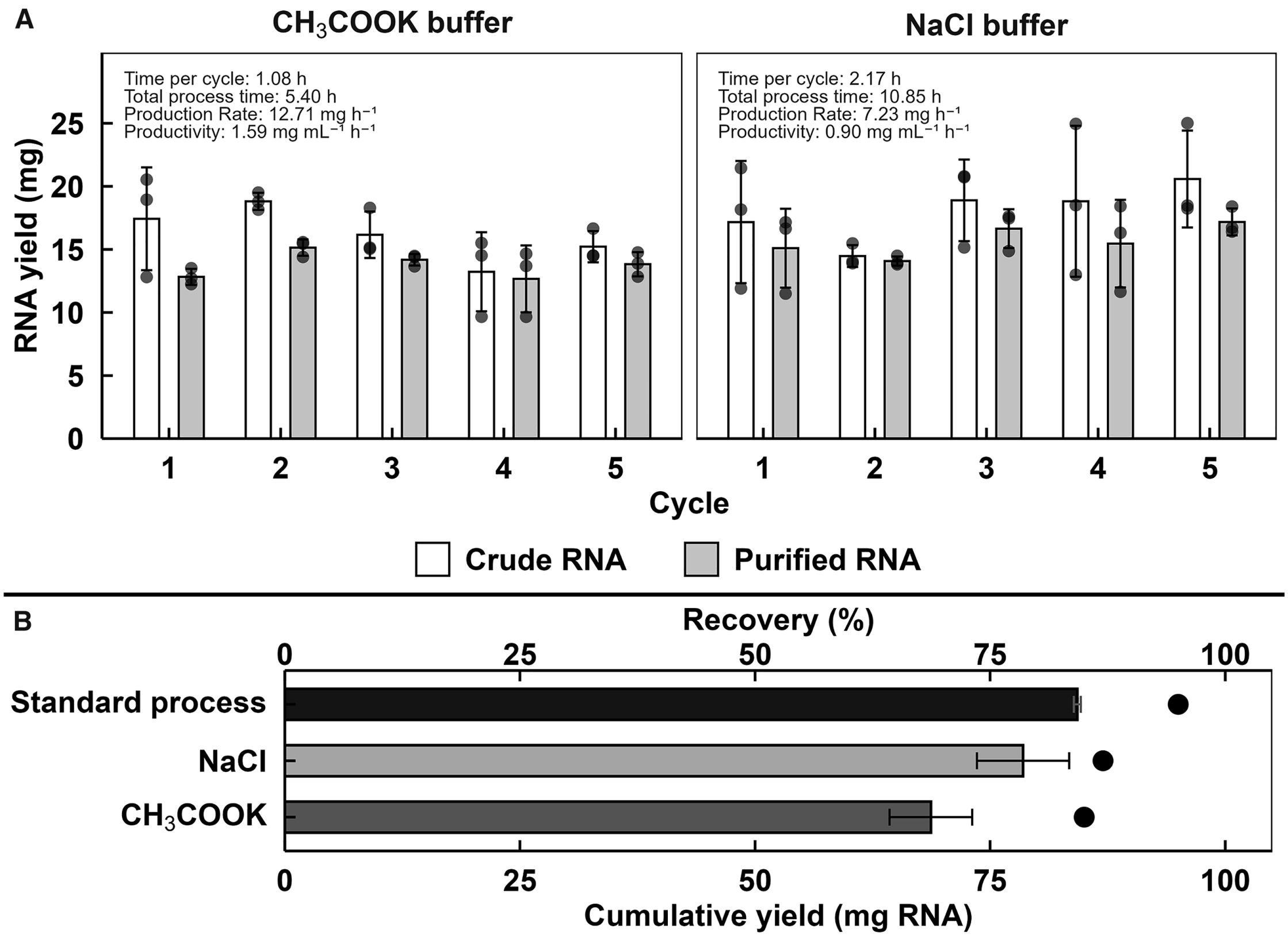

Figure 2. RNA yield and productivity across recycling cycles

(A) Crude and oligo-dT-purified RNA yields across five sequential cycles using NaCl- or CH_3COOK-based buffers. Annotations show cycle duration, total process time, production rate, and volumetric productivity. Bars show mean (SD), and points denote individual experiments ($n = 3$ independent experiments). (B) Cumulative purified RNA yield and percentage recovery after oligo-dT purification for the recycling workflows and standard batch IVT process. Horizontal bars show mean yield ± SD, and black points show mean recovery ($n = 3$ independent experiments). Kruskal-Wallis tests assessed cycle effects on purified RNA yield within each buffer in (A), and two-sided Welch's *t* tests were used for cumulative-yield comparisons in (B). See also Figure S1.

multiple recycling cycles while operating under buffer conditions compatible with both transcription and affinity capture. However, the standard process achieved higher space-time productivity by generating cumulative RNA mass comparable with the NaCl workflow and greater than the CH_3COOK workflow within a shorter total process time. The advantage of the integrated recycling workflow therefore lies primarily in reagent reuse and raw-material efficiency, rather than maximum short-term productivity. Having established the process-performance trade-off, we next examined how recycling affects raw-material utilization and overall production costs, which are key economic drivers of RNA manufacturing.

Economic impact: Efficiency in raw material utilization and cost improvements

Having established that the recycling process operates using either NaCl or CH_3COOK, we quantified raw-material input efficiency and cost relative to the standard process. As shown in Figure 3A, recycling improved 5′ cap-analog input efficiency by 3.72-fold with NaCl and 3.26-fold with CH_3COOK and improved the calculated input-normalized RNA output per amount of T7 RNA polymerase charged by 1.24-fold and 1.09-fold, respectively. By contrast, the calculated input efficiency of the DNA template and NTPs decreased: additional NTP input and predefined DNA supplementation were required across the five-cycle workflow relative to the cumulative purified RNA recovered. The lower calculated input-normalized template utilization therefore primarily reflects the greater cumulative DNA charge relative to purified RNA output. Template recovery in the recycled flowthrough was not directly quantified. Partial adsorption of plasmid DNA to the oligo-dT membrane or associated surfaces may also have reduced template carryover, but this remains unconfirmed. Despite these trade-offs, the calculated four-component raw-material cost decreased from USD 41.8/mg of purified RNA for the standard process to USD 18.9/mg with NaCl and USD 21.6/mg with CH_3COOK, corresponding to standard-to-buffer cost ratios of 2.21 and 1.94 and cost reductions of 54.8% and 48.3%, respectively (Figure 3B). Figure 3C decomposes this cost into four major raw-material contributions. Because the two buffer conditions received identical cumulative material inputs, their cost compositions were identical. Although the 5′ cap analog remained the largest contributor, its share decreased from 89% in the standard process

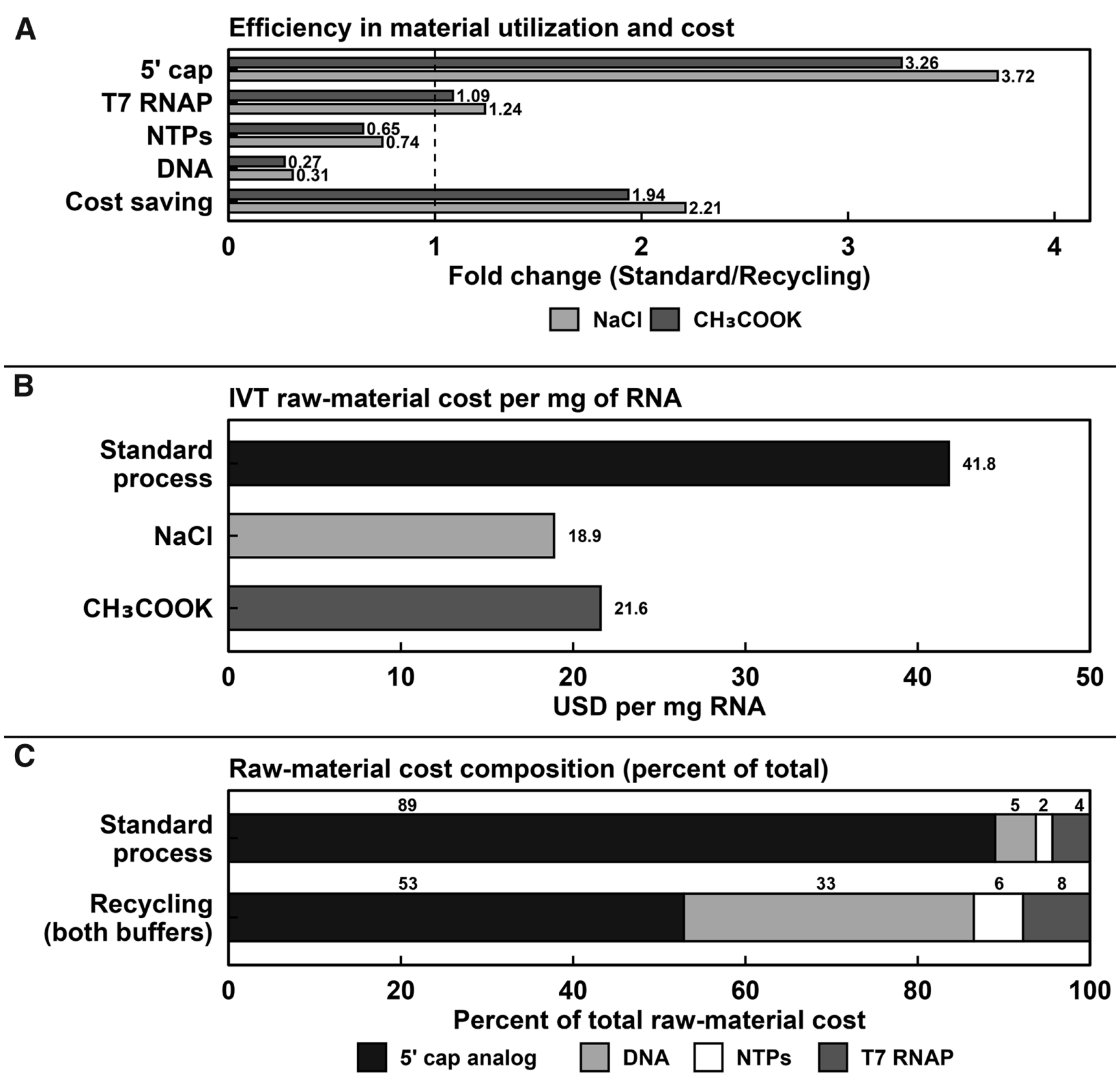

Figure 3. Raw-material utilization and cost analysis
(A) Standard-to-recycling ratios of the amounts of 5′ cap analog, T7 RNA polymerase (T7 RNAP), nucleotide triphosphates (NTPs), and DNA template charged per milligram of purified RNA and of the four-component raw-material cost per milligram of purified RNA for the NaCl and CH_3COOK recycling workflows. Each ratio was calculated as the standard-process input or cost per milligram of purified RNA divided by the corresponding recycling-process value. The dashed line at 1 denotes parity; values >1 denote lower input or cost requirements with recycling, and values <1 denote higher requirements with recycling. (B) Calculated four-component raw-material cost per milligram of purified RNA for the standard process and both recycling workflows. (C) Percentage contributions of the 5′ cap analog, DNA template, NTPs, and T7 RNAP to the four-component raw-material cost. A single recycling composition is shown because the two recycling workflows received identical cumulative material inputs. Calculations used mean purified RNA output from three independent experiments per condition, cumulative over five cycles for the recycling workflows. Source data and calculations are provided in Table S5.

to ∼53% during recycling. In parallel, the greater cumulative DNA charge increased the relative contribution of DNA template cost from 4.7% to 33.7%. Overall, the recycling workflow reduced the calculated four-component raw-material cost per milligram of purified RNA by approximately one-half under the demonstrated 8 mL laboratory-scale conditions and stated unit-price assumptions; this calculation is not a manufacturing-scale cost-of-goods estimate.

Product quality: RNA integrity, poly(A) tail heterogeneity, and sequence identity

While the recycling workflow improves reagent utilization and reduces raw-material cost, it is essential to verify that these gains do not compromise RNA quality. We therefore next evaluated key critical quality attributes (CQAs) of the resulting RNA, including RNA integrity, poly(A) tail heterogeneity, and sequence identity. RNA integrity was measured by capillary gel electrophoresis (CGE) and reported as the percentage of full-length transcripts. Across cycles, purified RNA consistently exhibited higher apparent integrity than crude RNA for both buffers. With NaCl, crude integrity averaged 88.2% ± 0.6%, increasing to 97.9% ± 0.3% after purification; with CH_3COOK, crude and purified integrity averaged 86.5% ± 0.6% and 98.0% ± 0.6%, respectively. In the standard process, crude and purified RNA integrity averaged 90.0% ± 2.0% and 96.3% ± 1.5%, respectively. The crude-purified difference likely reflects the removal of residual DNA and other non-full-length species from crude fractions, which lowers apparent integrity prior to purification, rather than restoration of individual RNA transcripts. Cycle-wise integrity trends and the standard-process comparator are shown in Figure 4A, with representative electropherograms in Figure S3. In addition, representative electropherograms of purified CSP mRNA from cycles 1 and 5 showed comparable electropherogram profiles (Figures S2B and S2C). Because crude CSP electropherograms were not available, these data are used as supportive evidence for preserved purified-product integrity in the longer transcript, rather than as a crude-to-purified integrity comparison.

Poly(A) tail heterogeneity was assessed for elution cycles 1 and 5 using the abundance-weighted deviation index (AWDI) and median tail-length statistics. In NaCl, both cycle 1 and cycle 5 showed AWDI = 0.01, a dominant species at ∼50 nt and median lengths of 53–54 nt (17.3–17.7 kDa), spanning 42–66 nt. CH_3COOK samples showed slightly broader heterogeneity (AWDI = 0.01–0.02) and shorter median lengths (43–47 nt, 14.1–15.6 kDa), with a similar modal species near 49–50 nt. No systematic differences were observed between cycles 1 and 5 within either buffer, indicating stable poly(A) distributions over repeated IVT-oligo-dT cycles. The standard process yielded median tail lengths of 55 nt and AWDI values of 0.01–0.02, consistent with run-to-run variability. Full summary statistics are provided in Table 1, and representative tail-length distributions for NaCl cycles 1 and 5, overlaid with the standard-process distribution, are shown in Figure 4B.

RNA sequence identity was evaluated for elution cycles 1 and 5 to confirm that recycling did not alter the primary sequence. Partial

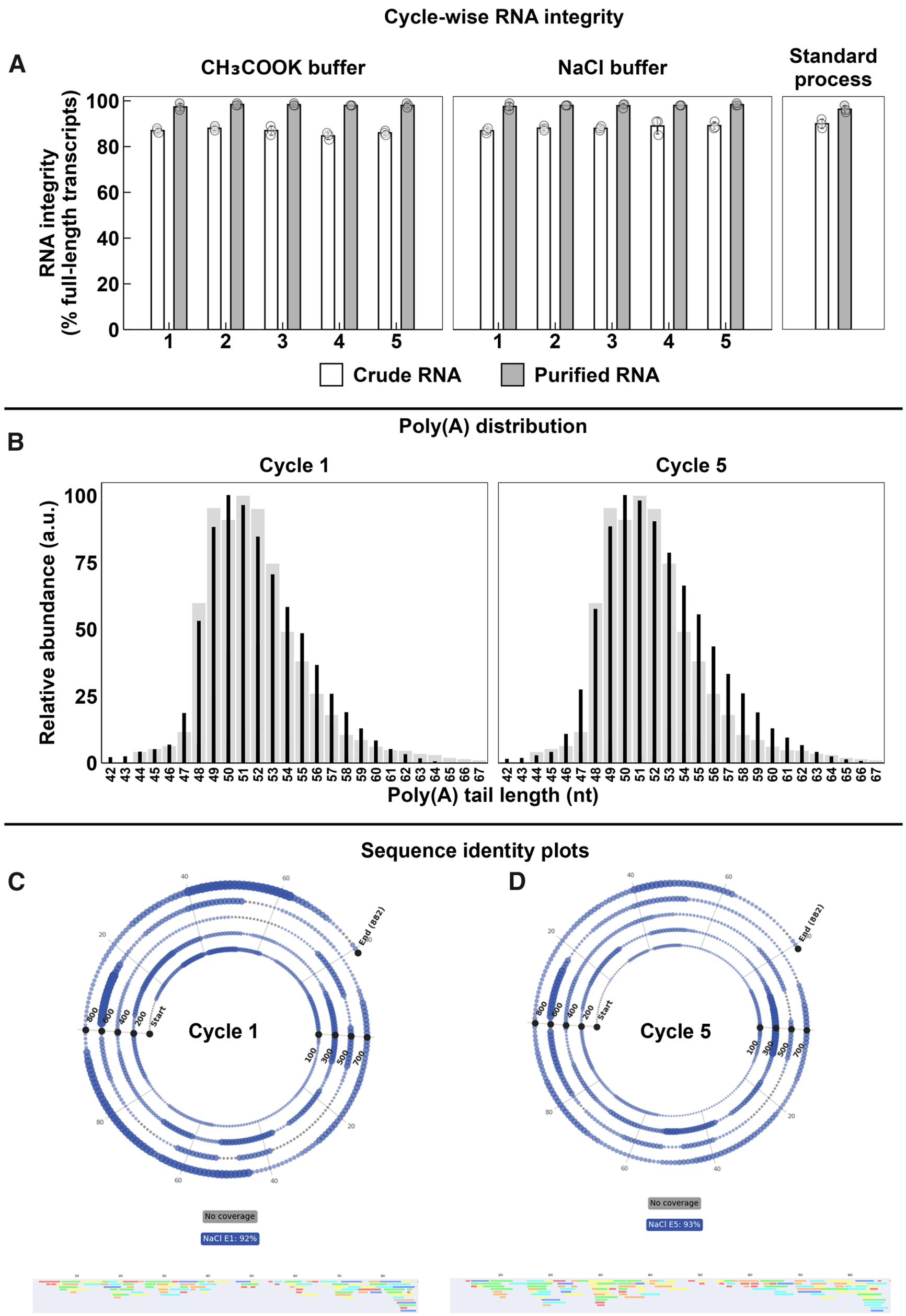

Figure 4. RNA integrity, poly(A)-tail profiles, and sequence identity

(A) Integrity of crude and oligo-dT-purified RNA across five IVT-oligo-dT recycling cycles using CH_3COOK- or NaCl-based buffers. Crude and purified RNA from the standard batch IVT process are shown in the separate right-hand images. Bars show mean (SD), and points denote individual experiments (n = 3 independent experiments per condition). (B) Representative poly(A) tail-length distributions for NaCl-derived RNA recovered after cycles 1 and 5, with the standard-process distribution overlaid in gray. Summary statistics are provided in Table 1. (C and D) Sequence-coverage maps for the 882-nt NaCl-derived RNA recovered after cycle 1 (C; 92% coverage) and cycle 5 (D; 93% coverage). Dot size and opacity reflect the summed confidence from overlapping mapped fragments; gray denotes no coverage. See also Figures S2 and S3.

RNase T1 and U2 digests were analyzed by mass spectrometry, and the resulting fragments were matched to the theoretical reference sequence. Cycles 1 and 5 showed 92% and 93% sequence coverage across the transcript, respectively, with no mismatched fragments detected within the covered regions, providing no evidence that recycling altered the primary sequence over successive cycles. The corresponding sequence identity plots are shown in Figures 4C and 4D.

Product quality: RNA 5′ capping efficiency anddsRNA content

CQAs such as 5′ capping efficiency and dsRNA content directly impact RNA function and are therefore central to therapeutic performance. These CQAs are particularly relevant to the recycling process because cap analog availability and transcription byproduct accumulation can change across cycles. We quantified 5′ capping by LC-MS across five IVT-oligo-dT cycles (Figure 5A). A downward trend was observed in both buffers: NaCl decreased from 86.3% ± 4.1% in cycle 1 to 75.7% ± 4.3% in cycle 5, and CH_3COOK decreased from 87.3% ± 8.2% to 62.3% ± 5.3%. Cycle 1 mean values were numerically comparable with those of the standard batch IVT process, suggesting that baseline performance was maintained initially. Although NaCl showed a numerical decline in capping efficiency across cycles, the cycle effect was not statistically significant at the tested sample size (Kruskal-Wallis, $H(4) = 5.47$, $p = 0.242$). In contrast, CH_3COOK showed a stronger cycle effect (Kruskal-Wallis, $H(4) = 12.6$, $p = 0.0136$), although no individual cycle pair remained significant after multiple-comparison correction (minimum adjusted q = 0.126). Because the five cycles are pooled to represent the cumulative five-cycle product, capping efficiency of the pooled product was calculated for each experiment as the yield-weighted mean across cycles and compared with the standard process. Pooled capping efficiency was 80.9% ± 4.2% for NaCl and 75.1% ± 1.8% for CH_3COOK, against 88.6% ± 1.3% for the standard process. The NaCl difference was not statistically significant ($\Delta = -7.73$ pp, 95% CI [−17.25, +1.80]; $p = 0.077$), whereas the CH_3COOK difference was ($\Delta = -13.47$ pp, 95% CI [−17.23, −9.71]; $p < .001$; two-sided Welch's t tests, $n = 3$ independent experiments).

The progressive reduction in capping efficiency reflects the one-time CleanCap AG charge used in the baseline workflow. CleanCap AG

Table 1. Poly(A) tail heterogeneity across IVT-oligo-dT cycles

Buffer	Cycle	Replicate	AWDI	Most abundant length (nt)	Median mass (Da)	Median length (nt)	Tail length range (nt)
NaCl	1	1	0.01	50	17365.8	53	42–64
		2	0.01	50	17493.9	53	42–64
		3	0.01	50	17658.9	54	43–65
	5	1	0.01	50	17386.3	53	42–66
		2	0.01	50	17493.9	53	42–65
		3	0.01	50	17329.8	53	43–64
CH_3COOK	1	1	0.02	50	14075.3	43	42–57
		2	0.02	50	15587.0	47	42–66
		3	0.02	49	15587.0	47	42–62
	5	1	0.01	50	15434.5	47	42–61
		2	0.02	49	15379.5	47	42–61
		3	0.02	50	15434.5	47	42–60
	–	1	0.01	51	18055.5	55	45–67
Standard process	–	2	0.01	50	18097.9	55	45–67
	–	3	0.02	50	18055.5	55	45–67

was added only in cycle 1 and was not replenished in cycles 2–5. Residual NTPs were not detected in NaCl eluates and ranged from 0.2% to 0.8% in CH_3COOK eluates across cycles (Table S4). The decline also paralleled the measured decrease in residual 5′ cap analog concentration in the flowthrough (Table S4), supporting reduced cap analog availability as a contributor to lower capping in subsequent transcription reactions. Thus, cap analog retention and controlled replenishment represent important control levers for maintaining capping efficiency during extended recycling. Despite the downward trend, the five-cycle mean capping efficiency remained above 80% in the NaCl workflow.

We next quantified dsRNA using a bioluminescent immunoassay across five recycling cycles and compared it with the standard batch IVT process (Figure 5B). dsRNA remained consistently low across cycles for both buffers. No cycle-to-cycle differences were detected (Kruskal-Wallis: NaCl, $H(4) = 4.87$, $p = 0.301$; CH_3COOK, $H(4) = 5.59$, $p = 0.232$), and no individual cycle pair was significant after correction (minimum adjusted q = 1.000). Yield-weighted pooled dsRNA content was 0.41% ± 0.18% for NaCl and 0.56% ± 0.10% for CH_3COOK, against 1.81% ± 0.55% for the standard batch IVT process, corresponding to 77.1% and 69.3% lower dsRNA, respectively. The NaCl difference was statistically significant ($\Delta = -1.40$ pp, 95% CI [−2.62, −0.17]; $p = 0.038$), while the CH_3COOK difference did not reach the significance threshold ($\Delta = -1.25$ pp, 95% CI [−2.57, +0.06]; $p = 0.055$; two-sided Welch's t tests, $n = 3$ independent experiments). Together, these data show that dsRNA did not increase progressively across recycling cycles and that oligo-dT-purified RNA from both recycling workflows contained lower dsRNA than oligo-dT-purified RNA from the standard batch IVT process. Because dsRNA was quantified after oligo-dT purification, these measurements do not distinguish whether the difference arose from altered dsRNA formation during IVT, differential impurity clearance during purification, or both.[31,32]

Functional performance: Protein expression and immunogenicity of recycled mRNA

Although analytical measurements provide detailed information on RNA CQAs, it is also important to confirm that recycled RNA performs as expected in a biological context. We therefore evaluated the functional activity and innate immune-stimulatory potential of the mRNA using THP-1 cell-based assays, which provide complementary readouts of translational output and innate immune activation. These assays were conducted using RNA samples for which dsRNA content and 5′ capping efficiency had already been quantified, allowing the cell-based response to be interpreted alongside the measured analytical CQAs. All IVT reactions used canonical UTP, providing an unmodified RNA context for assessing whether recycling was associated with progressive changes in protein expression or innate immune signaling.

mRNA produced using the NaCl buffer yielded a consistently higher percentage of enhanced green fluorescent protein (eGFP)-positive cells across all five cycles than both the CH_3COOK and standard batch IVT processes. Expressed as the yield-weighted pooled product, NaCl-derived mRNA gave 46.3% ± 4.5% eGFP-positive cells versus 26.7% ± 2.8% for the standard process ($\Delta = +19.55$ pp, 95% CI [+10.31, +28.79]; $p = 0.0057$), whereas CH_3COOK-derived mRNA did not differ from the standard process (26.4% ± 2.2%; $\Delta = -0.27$ pp, 95% CI [−6.03, +5.50]; $p = 0.90$; two-sided Welch's t tests, $n = 3$ independent experiments). No cycle-to-cycle drift was evident within either buffer (Kruskal-Wallis: NaCl, $H(4) = 2.01$, $p = 0.734$; CH_3COOK, $H(4) = 1.17$, $p = 0.884$; Figure 6A). Where available, fluorescence intensity measurements were also used to

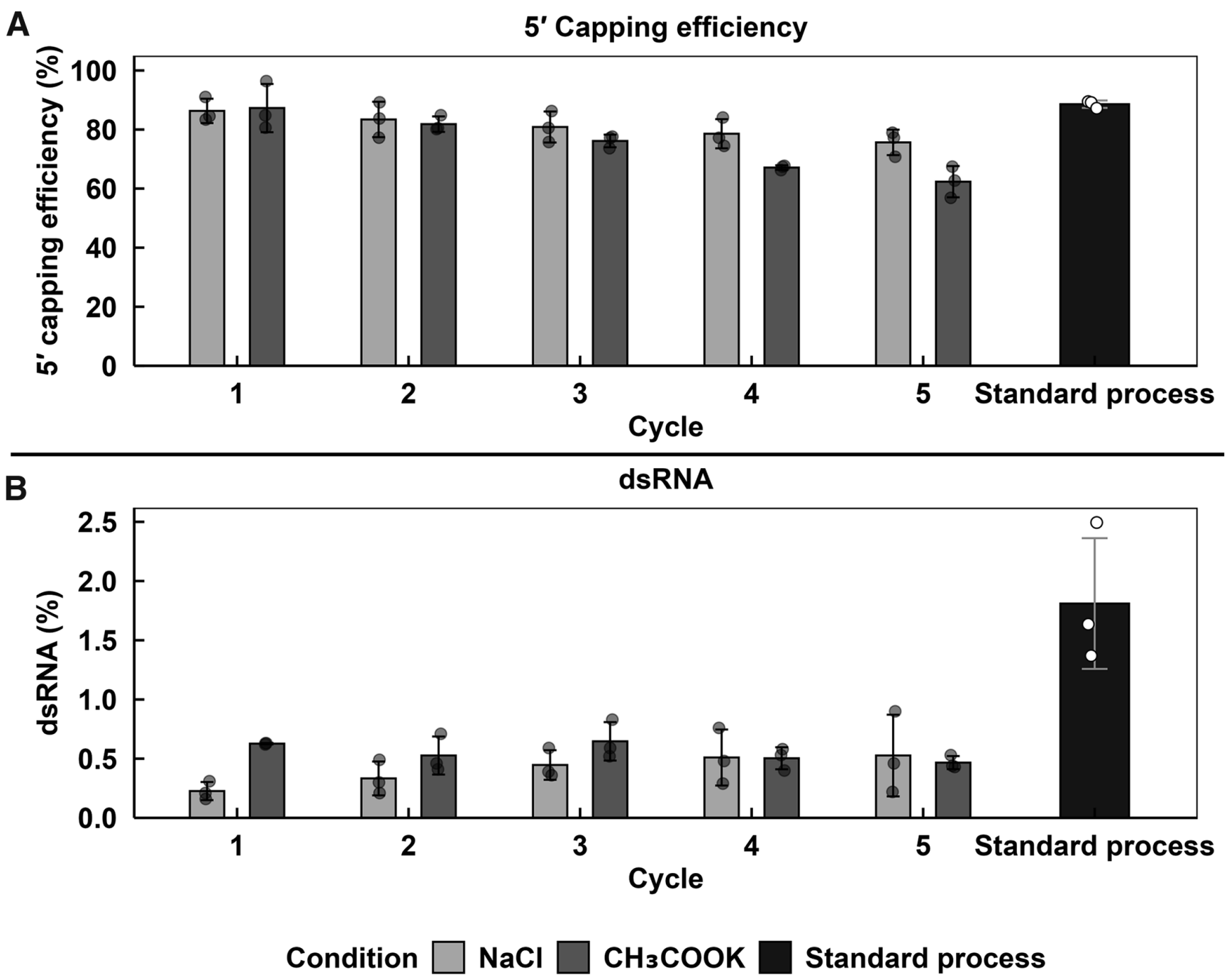

Figure 5. 5′ capping efficiency and dsRNA content
(A) 5′ capping efficiency across five IVT-oligo-dT recycling cycles using NaCl- or CH_3COOK-based buffers and for the standard batch IVT process. (B) dsRNA content across the same conditions. Bars show mean (SD); closed circles denote recycling measurements, and open circles denote standard-process measurements (n = 3 independent experiments). Kruskal-Wallis tests assessed cycle effects within each buffer, and two-sided Welch's t tests compared the yield-weighted pooled product of each buffer with the standard process. See also Table S4.

assess expression level per GFP-positive cell, complementing the percentage of eGFP-positive cells. Representative flow cytometry gating plots are shown in Figure 6B. Median GFP fluorescence intensity provided a complementary expression readout and showed higher GFP signal for NaCl-derived mRNA than CH_3COOK-derived mRNA across recycling cycles (Figure S4).

Cytokine measurements for the NaCl- and CH_3COOK-derived mRNA sample sets were acquired separately, and $\log_2$ fold changes for each sample set were calculated relative to the arithmetic mean of the standard-process measurements associated with it. Because each fold change is referenced to an estimated control mean specific to its sample set, the cytokine comparisons are reported descriptively, with reproducibility of the direction of change across the three independent experiments used in place of significance testing.

Relative to its corresponding standard-process comparator, NaCl-derived mRNA showed higher CCL3, CXCL10, and IFN-β (median $\log_2$ fold changes 2.37, 0.70, and 1.43, respectively), with each direction reproduced in all three independent experiments, whereas CXCL8, CCL5, IL-1β, and IFN-α showed no consistent shift. CH_3COOK-derived mRNA showed a different pattern relative to its own comparator, with higher CCL3 and IL-1β (0.28 and 0.17) and lower CXCL10, CXCL8, CCL5, and IFN-α (−1.32 to −0.13); IFN-β showed no consistent shift (Figure 6C). Importantly, no progressive increase in cytokine secretion was apparent across the five recycling rounds for either buffer, providing no evidence of cumulative innate immune activation with repeated reuse.

Together, these functional assays support maintenance of functional mRNA performance across cycles while revealing distinct cytokine-response patterns relative to the corresponding standard-process comparators. The higher eGFP expression observed with NaCl cannot be attributed to a single measured CQA alone, because the NaCl condition combined lower dsRNA content, higher overall 5′ capping than CH_3COOK, despite a decline across cycles, and preserved RNA integrity and poly(A)-tail profiles. Similarly, the higher CCL3, CXCL10, and IFN-β responses observed with NaCl relative to its corresponding standard-process comparator suggest that dsRNA content alone does not fully explain the innate immune profile. These data therefore support the functional feasibility of the integrated recycling workflow, while identifying buffer composition and the combined CQA profile as important variables for further optimization.

DISCUSSION

The continued expansion of RNA-based vaccines and therapeutics depends on manufacturing processes that are scalable, multi-product, cost-efficient, and resource-efficient. While recent advances have refined individual manufacturing steps, many efforts have addressed either process KPIs or product CQAs in isolation. This study integrates process development with comprehensive product characterization to advance reagent-recycling-based mRNA DS manufacturing. By considering upstream-downstream interactions rather than optimizing IVT and purification as independent unit operations, it provides an experimentally validated framework for connected RNA production. Previous studies have optimized IVT to achieve high conversion efficiency and reduced reagent usage, yet these advances were rarely accompanied by detailed RNA characterization or purification data.[33–36] Conversely, work that prioritized product quality was often conducted in narrowly defined settings with limited attention to process scalability.[37–39] A similar imbalance extends to downstream purification, where filtration or chromatography optimization has typically focused on isolated yield and recovery rather than overall process economics and reagent utilization.[22,29,30] The approach described

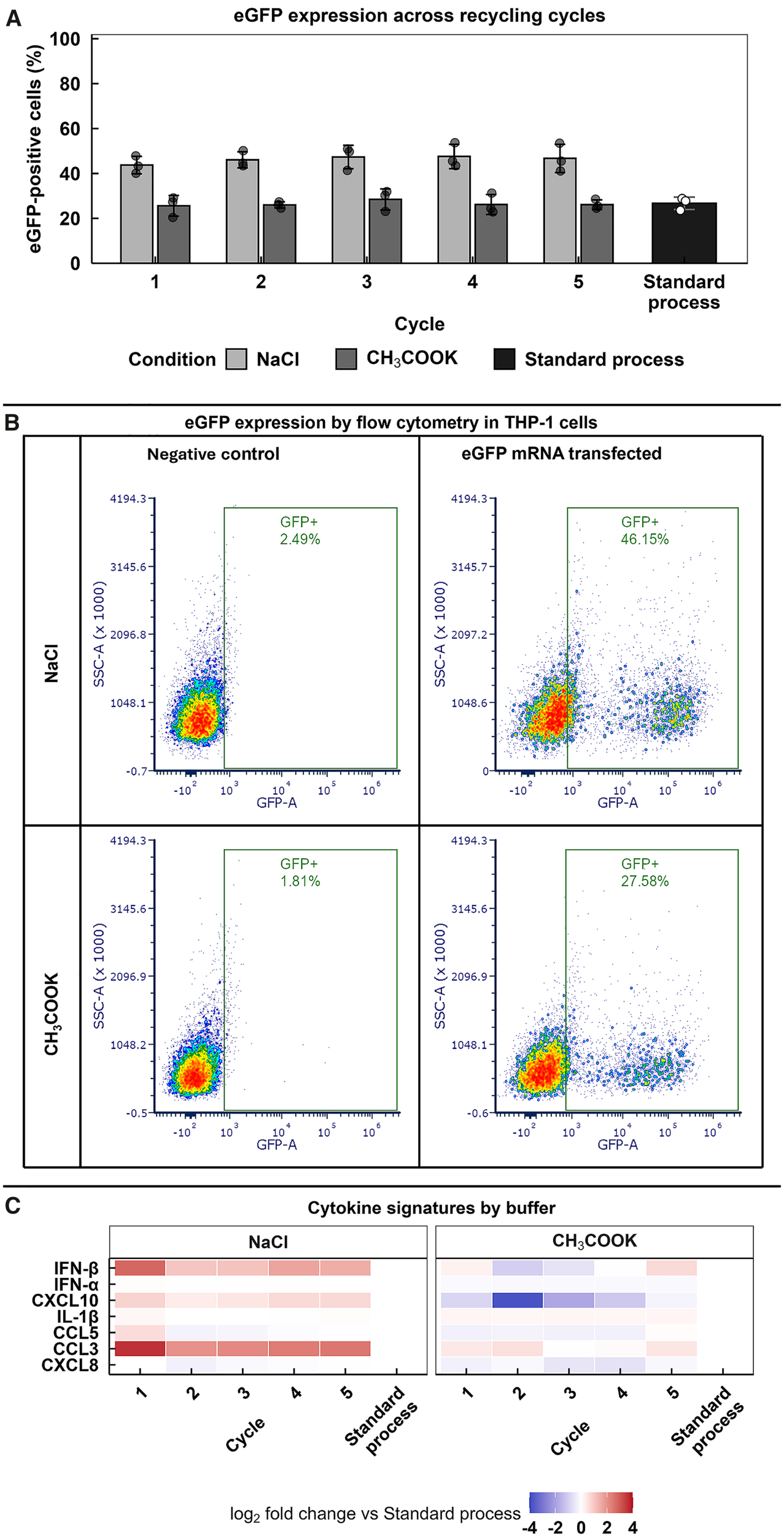

Figure 6. eGFP expression and cytokine responses in THP-1 cells

(A) Percentage of eGFP-positive THP-1 cells following transfection with mRNA from five NaCl- or CH_3COOK-based recycling cycles or from the standard batch IVT process. Bars show mean (SD); closed circles denote recycling measurements, and open circles denote standard-process measurements (n = 3 independent experiments). Kruskal-Wallis tests assessed cycle effects within each buffer, and two-sided Welch's t tests compared the yield-weighted pooled product of each buffer with the standard process. (B) Representative flow cytometry plots showing GFP-A on the x axis and SSC-A on the y axis for the negative control (untreated cells) and cells transfected with NaCl- or CH_3COOK-derived recycled mRNA. The GFP-positive threshold was defined using the untreated controls. (C) Cytokine secretion across cycles 1–5. The NaCl- and CH_3COOK-derived samples were evaluated as separate sample sets, and values are shown as mean log_2 fold change relative to the arithmetic mean of the corresponding standard-process measurements (n = 3 independent experiments). Each standard-process tile shows the mean of the individual standard-process measurements expressed on the same scale. Because replicate-level log_2 ratios are averaged after normalization to an arithmetic mean, these values are close to, but not constrained to, zero; the deviation is most visible for CXCL10. Sample-set-specific standard-process means were used for normalization. Values are presented descriptively, and no inferential significance testing was applied. Negative log_2 fold-change values indicate lower secretion than the corresponding standard-process control and should not be interpreted relative to untreated cells. See also Figure S4.

here bridges these perspectives by coupling process performance, raw-material utilization, and CQA assessment within a single integrated workflow.

Prior innovations have addressed several challenges examined here, particularly reducing 5′ cap analog usage and immunogenic byproducts.[37,39] Mutant T7 RNA polymerases can achieve high capping efficiency at substantially lower cap analog concentrations, and several variants are now commercially available, including GMP formulations. Engineered or alternative bacteriophage-derived RNA polymerases have also been reported to generate fewer immunogenic byproducts, such as dsRNA, potentially reducing the need for extensive downstream purification and associated costs. However, their transcriptional fidelity relative to the wild-type enzyme remains to be fully assessed.[38,40] Because the 5′ cap analog remains a dominant cost driver, other approaches have explored enzymatic capping strategies that bypass proprietary co-transcriptional cap analogs. These methods, however, introduce additional complexity through extra enzymatic steps or fusion proteins capable of both capping and transcription.[41,42] In practice, these strategies often substitute one proprietary reagent or process dependency for another and require techno-economic evaluation to confirm net advantage. Advanced purification techniques, including diverse chromatographic formats, have improved RNA CQAs[23,24,26] but have largely targeted impurity removal rather than recovery of unused IVT reagents. The distinctive contribution of the present work is therefore not simply higher IVT yield or improved purification, but the recovery and reuse of unconsumed high-value IVT components after product capture.

The cost advantage of the integrated process arises from how conventional high-yield IVT reactions are typically designed. In conventional high-yield IVT, NTPs are used at high concentrations, typically 7.5–15 mM per nucleotide.[33,36] Similarly high concentrations of 5′ cap analog are required to maintain efficient co-transcriptional capping because the cap analog competes with GTP during transcription initiation, with optimal cap-to-GTP ratios of 0.8–1.0.[15] As a result, although only one cap analog molecule is incorporated per RNA transcript, the reaction begins with a large excess of this reagent. This creates a major inefficiency: most of the input cap analog remains unconsumed after transcription and is normally discarded during purification. By enabling direct crude-IVT loading onto oligo-dT chromatography and recycling the flowthrough as the reaction matrix for subsequent IVT cycles, the integrated workflow converts this normally discarded stream into a reusable process input. This explains the improved cap analog utilization and raw-material cost reduction observed across the recycling workflow. Cap-analog mass balance indicates that this depletion is largely process-related rather than consumption-related. Because approximately one cap analog molecule is incorporated per transcript, five-cycle cumulative incorporation accounts for about 1% of the 20 μmol charged in cycle 1, whereas the residual cap analog concentration in the pooled flowthrough falls to 36.9% (NaCl) and 39.4% (CH_3COOK) of the cycle 1 value by cycle 5 (Table S4). Since flowthrough fractions were pooled on UV signal rather than as a fixed share of the load and wash volume, the cap analog leaving the recycle stream is consistent with incomplete recovery of unbound material during washing rather than with incorporation into transcript. Increasing the recovered fraction of the load-and-wash volume, or reducing wash volume at constant impurity clearance, is therefore a direct lever for sustaining capping efficiency across cycles without additional cap input. Extended recycling could employ either a validated predefined cap-analog addition for each cycle or residual-cap-guided supplementation coordinated with NTP control to maintain an appropriate cap-analog-to-initiating-NTP ratio. Neither strategy was evaluated in the present study. Both would increase total cap input and would therefore reduce the reported cap-utilization and raw-material-cost advantages.

The process also introduces a clear trade-off. The standard batch IVT process had higher short-term volumetric productivity because it generated cumulative RNA mass comparable with the NaCl workflow and greater than the CH_3COOK workflow in a shorter total process time. However, the 0.41 h CH_3COOK and 1.5 h NaCl durations were empirically selected operating points rather than matched kinetic endpoints. Comparisons therefore describe the configured workflows and should not be interpreted as intrinsic buffer effects at equivalent reaction completion. The integrated recycling workflow should not be interpreted as a productivity-maximizing replacement for high-yield batch IVT. Its advantage lies primarily in raw-material efficiency, cap analog utilization, and reduced reagent loss rather than maximum short-term throughput. This distinction is important for process positioning. In settings where facility time is the dominant constraint, the standard batch IVT process may remain preferable. In settings where cap analog cost, reagent availability, or raw-material sustainability dominates, the integrated recycling workflow becomes more attractive. Future techno-economic analyses should therefore evaluate the workflow under different manufacturing scenarios, including reagent-constrained, facility-constrained, and cost-constrained production settings. Under the demonstrated 8 mL conditions and stated unit-price assumptions, calculated raw-material cost per milligram of purified RNA was 54.8% lower for NaCl and 48.3% lower for CH_3COOK than for the standard process, corresponding to 2.21- and 1.94-fold improvements in raw-material cost efficiency, respectively. These values are not manufacturing-scale cost-of-goods estimates. Translation to manufacturing scale will depend on scale-dependent reagent pricing, recovery, automation, labor and facility utilization, quality-control and validation requirements, and oligo-dT device capacity and lifetime.

The lower dsRNA content measured in purified RNA from the recycling workflows should be interpreted as a product-quality outcome of the complete IVT-oligo-dT process rather than direct evidence that recycling reduced dsRNA formation during transcription. The observed difference could reflect altered IVT conditions, including lower per-cycle RNA accumulation, shorter product residence time and elevated ionic strength; differential impurity partitioning during oligo-dT capture; or a combination of these effects. Specific retention on oligo-dT is governed primarily by the presence and accessibility of a poly(A) tail. Abortive or truncated transcripts

that terminate before the template-encoded poly(A) sequence would therefore be expected predominantly in the flowthrough or wash, whereas truncated or aberrant species retaining a sufficiently long poly(A) tail may bind and co-elute with the product. These RNA-impurity populations were not directly quantified in the recycled flowthrough. Purified-product yield, integrity, and dsRNA content did not deteriorate over five cycles. However, these endpoints were not designed to quantify RNA impurities in the recycled flowthrough and therefore neither demonstrate nor exclude their accumulation or competitive effects on subsequent transcription. A dedicated impurity-partitioning study should therefore compare matrix-matched pre- and post-oligo-dT samples and characterize the flowthrough for RNA size distribution, poly(A) status, and dsRNA content. Previous studies provide plausible mechanisms by which lower RNA concentration, shorter product residence time, and elevated ionic strength may reduce RNA-templated secondary transcription; however, these mechanisms were not independently deconvoluted in the present workflow.[31,32,43–46] High salt also enables direct capture on oligo-dT by shielding electrostatic repulsion between oligo-dT ligands and the poly(A) tail, thereby enhancing binding.[28] Preliminary time-course screening showed that NaCl and CH_3COOK produced different apparent transcription kinetics under the shared-buffer conditions, consistent with reports that solution composition and specific ions affect T7 RNAP activity.[36,47] RNA accumulation was limited to maintain manageable viscosity and minimize poly(A)-RNA breakthrough into the recycled flowthrough. Under the tested conditions, these results identify three process-design implications for integrated RNA manufacturing: (1) moderate substrate use across multiple cycles can improve raw-material efficiency while maintaining a broad CQA profile; (2) lower per-cycle RNA accumulation and shorter product residence time may limit RNA-templated secondary transcription while keeping viscosity compatible with direct column loading; and (3) elevated ionic strength can support oligo-dT capture while altering the transcription environment and potentially influencing the final purified-product dsRNA profile. Thus, the shared buffer is not a passive medium but a coupled process variable that affects transcription, capture, recycle-stream composition, and product quality.

The THP-1 assays further demonstrate that biological performance was not predicted by any single measured CQA. NaCl-derived mRNA produced a higher proportion of eGFP-positive cells than CH_3COOK-derived and standard-process mRNA, despite showing lower capping than the standard process, and, relative to its corresponding standard-process comparator, induced higher CCL3, CXCL10, and IFN-β secretion. Within their respective assay series, NaCl- and CH_3COOK-derived mRNA showed distinct cytokine-response patterns relative to the corresponding standard-process controls. Because the buffer arms were evaluated in separate assay series and differed simultaneously in capping, dsRNA content, and potentially unmeasured process-related attributes, these patterns cannot be attributed to buffer identity, dsRNA, or capping alone. No progressive cycle-dependent drift in eGFP expression or increase in cytokine secretion was observed, providing no evidence of cumulative functional deterioration or innate immune activation over the five-cycle operating window.

The main limitations arise from an inherent trade-off: the recycling-based process balances two coupled unit operations rather than allowing each to operate at its individual optimum. This compromise improves raw-material efficiency and preserves a broad CQA profile, but it reduces short-term productivity relative to the standard batch IVT process. When RNA concentration becomes too high, viscosity increases, hindering flow and promoting column clogging; conversely, the ionic strength required for oligo-dT binding can inhibit transcription. Several strategies may mitigate these constraints. Modified promoters used with wild-type T7 can permit operation at elevated salt concentrations without substantial kinetic penalties. Nicked promoters or single-stranded templates can improve initiation and transition to elongation, increasing tolerance to higher ionic strength.[43,48] From the purification side, improving column hydrodynamics by using geometries that reduce pressure drop while maintaining capacity, such as monoliths or fibrous adsorbents, could enhance efficiency and scalability.[28,30] The oligo-dT membrane used here was selected for proof-of-concept development because it enabled direct crude-IVT loading, rapid product capture, and straightforward recovery of the flowthrough for reuse. The membrane and operating load were chosen to reduce the risk of poly(A)-RNA breakthrough into the recycle stream rather than to maximize chromatographic capacity utilization, because uncaptured RNA would otherwise be carried into the subsequent IVT cycle. Manufacturer-reported binding capacities obtained under conventional oligo-dT conditions are not directly transferable to the crude-IVT shared-buffer matrices used here. Process-specific breakthrough, pressure-flow, capacity, and device-lifetime studies under the actual recycling conditions will therefore be required for scale-up. A breakthrough curve and formal dynamic binding capacity were not measured, and the observed product recovery should therefore not be interpreted as a capacity determination.

The present configuration is a laboratory-scale, sequential batch recycling process, not a simultaneous reaction-separation system or continuous manufacturing process. In this study, integration denotes chemical and operational coupling through shared-buffer compatibility, direct crude-IVT loading, oligo-dT capture, and reuse of the chromatography flowthrough between successive batch IVTs. This definition is distinct from simultaneous reaction-separation coupling or closed-loop continuous operation. The current process establishes a foundation for future automated or continuous IVT-purification architectures by demonstrating that upstream transcription and downstream capture chemistries can be linked through a common solution environment. Further development could automate flowthrough collection, inline supplementation, and recycle handling to reduce manual operations and improve reproducibility. Tighter control of column equilibration and switching, including reduced hold-up and axial dispersion during loading, washing, and elution, would also reduce unintended mixing of the crude IVT

stream with column buffers. Losses of key reagents, such as template DNA and T7 RNA polymerase, could be reduced by improved surface compatibility or immobilization strategies, supporting more efficient reagent reuse and eventual transition toward continuous RNA synthesis.[27,49] Although feasibility was assessed with a longer CSP transcript, process efficiency was lower than for eGFP, presumably due to viscosity, precipitation, and loading constraints that are commonly more pronounced with larger mRNA transcripts.[28] A broader evaluation across templates and a mechanistic dissection of innate immune responses, including contributions of dsRNA, incomplete 5′ capping, nucleotide chemistry, and residual process-related impurities, were beyond the scope but remain active areas for optimization. Nevertheless, the combined KPI, CQA, and functional assay data support the feasibility of the integrated recycling workflow and identify candidate control points for translation beyond laboratory-scale proof of concept. Translation toward GMP-relevant implementation will require a defined control strategy. In the demonstrated workflow, NTPs and $MgCl_2$ were replenished using fixed cycle-wise additions, while a separate predefined template-DNA top-up was intended to compensate for expected process loss. These additions were not determined from cycle-specific measurements of residual NTPs, free Mg^{2+}, or template-DNA recovery. Cap analog concentration in the flowthrough and residual rNTPs in the purified eluate were quantified. Residual NTP concentrations in the recycled flowthrough, total and free Mg^{2+}, PPi concentration, residual PPase activity, and T7 RNA polymerase concentration and catalytic activity were not directly quantified across cycles. Exogenous PPase was included in the IVT formulation. The absence of a progressive decline in per-cycle RNA yield indicates that the combined effects of any PPi accumulation, Mg^{2+} limitation, or T7 RNA polymerase loss were insufficient to cause progressive yield deterioration over the five-cycle operating window. However, this process-level evidence does not establish the concentrations or activities of these components in the recycled flowthrough. PPi formation, magnesium pyrophosphate precipitation, and free Mg^{2+} availability are coupled during IVT, and direct monitoring of free Mg^{2+} can provide a more informative basis for assessing PPase performance and Mg^{2+} availability than nominal total Mg^{2+} concentration alone.[50,51] Extended recycling should therefore use predefined acceptance ranges and cycle-specific measurements for cap analog and NTP concentrations, total and free Mg^{2+}, PPi, residual PPase activity, T7 RNA polymerase concentration and catalytic activity, DNA template carryover, residual RNA impurities, conductivity, pH, and hold time. Cap analog could be replenished to a predefined cycle-start concentration and cap analog:GTP ratio using flowthrough measurements, while NTP and Mg^{2+} supplementation could be adjusted from measured residual concentrations rather than a fixed supplementation recipe. T7 RNA polymerase concentration could be monitored using a polymerase-specific capillary immunoassay, although an orthogonal activity assay would still be required to establish active enzyme recovery.[52] These measurements would enable feedback-controlled supplementation, distinguish component loss from activity decay, and define a maximum validated number of recycle cycles. For longer recycling campaigns, free PPase supplementation could be adjusted using PPi or free-Mg^{2+} measurements, while immobilized PPase could be evaluated as an alternative approach to maintain PPi hydrolysis and limit enzyme loss from the recycle stream. Because GuHCl was included in the shared buffer but was not varied independently, its specific contributions to transcription, loading behavior, impurity control, and recycle-stream stability remain to be established.

The platform scope is clearest for poly(A)-tailed mRNA because oligo-dT capture depends on poly(A)-oligo-dT hybridization. The CSP data support feasibility beyond the shorter eGFP construct, but broader transcript generality will require systematic evaluation across RNA lengths, sequences, nucleotide chemistries, and poly(A)-tail designs. Within poly(A)-tailed mRNA, transcript length and construct-specific sequence or secondary structure may affect IVT kinetics and byproduct formation.[36,45] Even though generality was assessed using a longer transcript, process efficiency was lower, potentially reflecting viscosity, precipitation, and loading constraints associated with larger mRNA transcripts.[28] Ongoing studies are systematically evaluating these variables across a broader transcript panel to define the operating range and transcript dependence of the recycling workflow. Polyadenylated saRNA could, in principle, use the same capture mechanism, although its greater length and structural complexity would require separate evaluation of IVT kinetics, viscosity, loading, and recovery. Extension to RNA modalities lacking a poly(A) tail, such as many sgRNAs or circRNAs, would require alternative capture ligands or engineered affinity handles and a new compatibility assessment for reaction chemistry, capture conditions, and recycle-stream composition. Thus, the present work should be positioned as an experimentally demonstrated integrated recycling strategy for poly(A)-tailed mRNA manufacturing and as a broader conceptual foundation for reagent-reuse strategies in RNA production.

In summary, the sequential batch IVT-oligo-dT workflow reduced calculated raw-material cost and improved cap analog utilization while maintaining stable cycle-wise RNA production and several measured CQAs across five recycling cycles. Oligo-dT-purified RNA retained high integrity and stable poly(A)-tail profiles, and sequencing of cycles 1 and 5 revealed no mismatches within the covered regions. Purified recycling-process RNA also contained less dsRNA than the purified standard-process comparator, although altered formation and differential clearance could not be distinguished. Functional expression was maintained without progressive cycle-wise drift. Declining capping efficiency, accompanying progressive cap analog depletion, remained the principal measured CQA limitation. These findings establish laboratory-scale proof of concept for resource-efficient reagent recycling and identify the measurements and control strategies required before extended, automated, or continuous implementation.

MATERIALS AND METHODS

Key reagents, materials, instruments, and software used in this study are listed in Table 2.

Table 2. Key resources table

Reagent or Resource	Source	Identifier
T7 RNA polymerase	Roche (Germany)	8140669103
RNase inhibitor	Roche (Germany)	9537589103
Inorganic pyrophosphatase	Roche (Germany)	8140677103
ATP	Roche (Germany)	9744410103
CTP	Roche (Germany)	9745122103
GTP	Roche (Germany)	9745220103
UTP	Roche (Germany)	9962689103
CleanCap AG	TriLink (USA)	N-7113
$MgCl_2$	Thermo Fisher Scientific (USA)	AM9530G
Tris-HCl	Thermo Fisher Scientific (USA)	AM9851
DTT	Thermo Fisher Scientific (USA)	R0861
Spermidine	Sigma-Aldrich (USA)	85558-5G
Triton X-100	Sigma-Aldrich (USA)	648466
NaCl	Thermo Fisher Scientific (USA)	AM9759
Potassium acetate (CH_3COOK)	Thermo Fisher Scientific (USA)	J60832.AK
Guanidine hydrochloride (GuHCl)	Sigma-Aldrich (USA)	50937-100ML
Silica RNA cleanup columns	NEB (USA)	T2050L
ÄKTA Pure 25	Cytiva (Sweden)	–
UNICORN software	Cytiva (Sweden)	v7.8
10 mL sample loop	Cytiva (Sweden)	–
Purexa OdT Maxi membrane column, 2 mL, 0.5 μm	Donaldson (USA)	–
DNAPac PA200 column (50 mm × 2.1 mm)	Thermo Fisher Scientific (USA)	–
DNAPac RP column (2.1 mm I.D.)	Thermo Fisher Scientific (USA)	–
UltiMate 3000 HPLC	Thermo Fisher Scientific (USA)	–
Chromeleon software	Thermo Fisher Scientific (USA)	v7.2.10
Fragment analyzer 5200	Agilent (USA)	–
DNF-471 RNA Kit (15 nt)	Agilent (USA)	–
FA 12-capillary array short, 33 cm	Agilent (USA)	–
ProSize software	Agilent (USA)	–
RNase T1	Thermo Fisher Scientific (USA)	EN0542
SMART Digest Bulk Magnetic RNase U2 Kit	Thermo Fisher Scientific (USA)	–
Triethylammonium acetate	Sigma-Aldrich (USA)	–
Vanquish UHPLC	Thermo Fisher Scientific (USA)	–
Orbitrap Exploris 240	Thermo Fisher Scientific (USA)	–
ACQUITY premier oligonucleotide C18 (2.1 × 100 mm, 1.7 μm, 130 Å)	Waters	–
Dibutylamine (DBA)	Laboratory stock	–
HFIP	Laboratory stock	–
Lumit dsRNA detection assay	Promega	W2041
THP-1 cells	Laboratory stock	–
RPMI-1640	Laboratory stock	–
FCS (heat-inactivated)	Laboratory stock	–
TransIT-mRNA transfection reagent	Mirus Bio (USA)	–
TransIT-mRNA boost reagent	Mirus Bio (USA)	–
Opti-MEM	Thermo Fisher Scientific (USA)	–

(*Continued on next page*)

Table 2. *Continued*

Reagent or Resource	Source	Identifier
LIVE/DEAD fixable violet dead cell stain	Thermo Fisher Scientific (USA)	–
Cytek Aurora 5-laser spectral flow cytometer	Cytek Biosciences (USA)	–
FCS Express	De Novo Software (USA)	–
BD CBA cytokine kits (IFN-α, IL-1β, CCL5, CCL3, CXCL10, CXCL8)	BD Biosciences (USA)	560379, 558279, 558324, 558325, 558280, 558277
BD CBA Master Buffer Kit	BD Biosciences (USA)	558265
IFN-β ELISA	R&D Systems (USA)	DY814-05
Tecan Infinite M200 plate reader	Tecan	–
GraphPad Prism	GraphPad	v10
R	R Core Team	v4.5.0
R packages: tidyverse, MSnbase, xcms, mzR, pracma, etc.	CRAN/Bioconductor	–

In vitro transcription

RNA transcripts were synthesized by IVT using a linear DNA template with a sequence described in Supplemental Information under "Template DNA sequences." The IVT mixture contained T7 RNA polymerase (Roche, Germany), ribonucleoside triphosphates (ATP, CTP, GTP, UTP; Roche, Germany), and the co-transcriptional 5′ cap analog CleanCap AG (TriLink, USA). Reactions were supplemented with magnesium chloride, Tris-HCl (pH 7.0), dithiothreitol (DTT) (all from Thermo Fisher Scientific, USA), spermidine (Sigma-Aldrich, USA), Triton X-100 (Sigma-Aldrich, USA) and, where indicated, with sodium chloride (NaCl; Thermo Fisher Scientific, USA) or potassium acetate (CH_3COOK; Thermo Fisher Scientific, USA) and guanidine hydrochloride (GuHCl; Sigma-Aldrich, USA). Inorganic pyrophosphatase (Roche, Germany) was added to hydrolyze PPi released during transcription and prevent magnesium pyrophosphate precipitation. RNase inhibitor (Roche, Germany) was included to maintain an RNase-free environment. Reactions were incubated at 37 °C for 1.5 h (NaCl-based) or 0.41 h (CH_3COOK-based). These durations were empirically selected process operating points rather than matched kinetic endpoints. For yield quantification following IVT, aliquots of crude IVT were cleaned up by solid-phase extraction on silica columns (NEB, USA) following the manufacturer's protocol to remove UV-interfering reagents and quantified by UV absorbance at 260 nm using a NanoDrop One^c spectrophotometer (Thermo Fisher Scientific, USA), normalized to a 1.0 cm path length. The complete compositions for the standard and integrated IVT-oligo-dT recycling processes are listed in Table S1. All main reactions were performed on an 8 mL scale.

Oligo-dT affinity chromatography

All oligo-dT affinity chromatography procedures were performed using an ÄKTA Pure 25 system (Cytiva, Sweden) equipped with dual binary pumps and four inlet valves (A1, A2, B1, and B2) to enable buffer selection. Absorbance was monitored at 260 nm using an integrated multi-wavelength UV detector, and conductivity was recorded in line to track salt concentration. Samples were injected using a 10 mL sample loop (Cytiva, Sweden). Method programming, system operation, and data analysis were performed using UNICORN software v7.8 (Cytiva, Sweden). A 2 mL Purexa OdT Maxi membrane column (0.5 μm pore size; Donaldson, USA) was used for oligo-dT affinity capture. The purification protocol comprised column equilibration, sample loading, washing, elution, and cleaning-in-place (CIP). The column was equilibrated with 10 column volumes (CV) of equilibration buffer, after which 8 mL of IVT reaction mixture was loaded under the same buffer conditions to promote poly(A)-oligo-dT hybridization. A wash step (10 CV) was performed to remove unbound and loosely bound components. Flowthrough fractions generated during loading and washing were monitored at 260 nm (typically 50–100 mAU) and collected in 96-deep-well plates (1 mL per fraction). These fractions (total ≈ 8 mL) were pooled and reused as the reaction matrix for subsequent IVT runs after supplementation with predefined NTP and $MgCl_2$ additions. Flowthrough supplementation was performed using the predefined NTP and $MgCl_2$ additions specified for each subsequent IVT cycle in Table S2. Supplementation was not adjusted using cycle-specific measurements of residual NTP or Mg^{2+}; therefore, true cycle-start concentrations were unknown. The fixed additions were predefined and were not calculated from cycle-specific RNA yield or a product-based mass balance. Template DNA was supplemented before cycles 2–5 at 50% of the cycle 1 input (200 μg per cycle), resulting in a cumulative template-DNA input of 1,200 μg across five cycles. Template supplementation was predefined and was not adjusted using cycle-specific measurements of residual DNA in the recycled flowthrough. CleanCap AG was not included in the between-cycle supplementation. It was charged only in cycle 1 at 2.5 mM (20 μmol), while cycles 2–5 used residual cap analog carried forward in the pooled flowthrough. Bound mRNA was eluted isocratically using nuclease-free water (10 CV), and the column was regenerated by CIP with 0.1 M NaOH (10 CV). Buffer compositions for the standard oligo-dT and integrated IVT-oligo-dT methods are provided in Table S3.

Quantification of unincorporated NTPs and 5′ cap analog with AEX-HPLC

The concentrations of the 5′ cap analog in the flowthrough and residual NTP contaminants in the elution from oligo-dT

chromatography were quantified using anion-exchange (AEX) HPLC on a U3000 system equipped with a DNAPac PA200 column (50 mm × 2.1 mm I.D.; Thermo Fisher Scientific, USA). The analysis followed the protocol of Welbourne et al.[53] Chromeleon software version 7.2.10 (Thermo Fisher Scientific, USA) was used to integrate peak areas and quantify against calibration curves for each NTP and the 5′ cap analog.

Quantification of RNA integrity with CGE

CGE measured RNA integrity (% full-length transcripts) of oligo-dT purified samples on a 5200 Fragment Analyzer System (Agilent, USA), which separates and detects RNA fragments based on size. The method employed the DNF-471 RNA Kit (15 nt) (Agilent, USA), which consists of an RNA separation gel, dsDNA inlet buffer, TE rinse buffer, intercalating dye, RNA diluent marker (15 nt), RNA ladder (200–6,000 nt), and capillary conditioning solution. An FA 12-Capillary Array Short, 33 cm (Agilent, USA), was used for the separations. Purified mRNA samples were diluted in nuclease-free water to ≈50 ng/μL and further diluted 1:12.5 in RNA diluent marker to a final concentration of ∼4 ng/μL. Before each run, a pre-run voltage (8 kV for 30 s) was applied, and the capillaries were conditioned with the conditioning solution and dipped twice in the rinse buffer. The capillaries were then filled with RNA separation gel (by pressure), and the sample was introduced by voltage injection (5 kV for 4 s). Separation was performed at 8 kV for 45 min. Detection was achieved by laser-induced fluorescence using a fluorescent dye incorporated into the separation gel. Data were analyzed with Agilent ProSize Data Analysis Software.

Quantification of 5′ capping efficiency with LC-MS

RNase digestion was performed on 25 μg RNA employing 60 U RNase T1 (Thermo Fisher Scientific) in 0.1 M triethylammonium acetate (Sigma-Aldrich) at 37 °C overnight to generate 5′ cap and poly(A) fragments. Before LC-MS, 10 mM EDTA (pH 8, adjusted with ammonium hydroxide) was added to minimize sodium adducts. Ion-pair reversed-phase (IP-RP) HPLC-UV-MS was carried out on a Vanquish UHPLC coupled to an Orbitrap Exploris 240 mass spectrometer (Thermo Fisher Scientific) using a Waters ACQUITY Premier Oligonucleotide C18 Column (130 Å, 1.7 μm, 2.1 × 100 mm) (Waters). Mobile phases comprised 10 mM dibutylamine (DBA) + 50 mM hexafluoro-2-propanol (HFIP) in water (A) and in 50% acetonitrile (B). Separations were performed at 50 °C with UV detection at 260 nm, starting with 5% B for 1 min, followed by a ramp to 35% B over 60 min. MS data were acquired in negative-ion mode (HESI source, 2.5 kV; sheath gas 35; aux gas 10; ion-transfer 320°C; vaporizer 350°C). MS1 scans (430–1,350 m/z, 120 k resolution) were followed by data-dependent HCD MS2 (150–1,500 m/z, 30 k resolution, 3 m/z isolation window). Capping efficiency was quantified using a custom R Shiny application and calculated based on Equation 1. Raw LC-MS data were converted to .mzML and processed with MSnbase to extract ion chromatograms (EICs) for user-defined capped and uncapped species. Retention-time ranges (±30 s default) were interactively selected in plotly plots, and peak areas integrated by trapezoidal rule (pracma), with optional MS2 confirmation of singly charged ions.

$$\text{Capping efficiency} = \frac{\sum \mathrm{AUC_{capped}}}{\sum \mathrm{AUC_{capped}} + \sum \mathrm{AUC_{uncapped}}} \times 100 \quad \text{(Equation 1)}$$

where

AUC_{capped} = integrated area under the EIC of the capped species

AUC_{uncapped} = integrated area under the EIC of the uncapped species

Quantification of poly(A) heterogeneity with LC-MS

Poly(A) tail fragments were analyzed by IP-RP HPLC-UV-MS on a Vanquish UHPLC coupled to an Orbitrap Exploris 240 mass spectrometer (Thermo Fisher Scientific). The gradient was initiated at 35% B, increased linearly to 40% B over 1 min, and then to 50% B over 30 min at a flow rate of 0.25 mL $\mathrm{min^{-1}}$. MS acquisition was performed using the intact protein method in low-pressure mode with a resolving power of 240,000, a scan range of 750–1,500 m/z, an RF lens of 75%, and a normalized AGC target of 100%. Maximum injection mode was set to custom with a 300 ms injection time, and five microscans were recorded in negative-ion mode. Poly(A) heterogeneity was quantified using a custom R Shiny application implementing the AWDI. Monoisotopic masses were matched to theoretical poly(A) monoisotopic masses at nucleotide resolution to determine tail lengths, and AWDI was computed by weighting squared deviations from the expected poly(A) tail mass by each species' relative abundance, yielding a single metric of heterogeneity. The expected mass is calculated from the poly(A)-tail length encoded in the template DNA. Retention-time, mass, and abundance filters were applied to suppress noise and improve quantitation.

$$\mathrm{AWDI} = \sum_{i=1}^{n} w_i \left(\frac{m_i - m_0}{m_0} \right)^2 \quad \text{(Equation 2)}$$

where

m_i = observed monoisotopic mass of the ith poly(A) species

m_0 = monoisotopic mass of the expected full-length poly(A) tail

w_i = fractional abundance of the ith species

n = number of fragments after filtering

Quantification of RNA sequence identity with LC-MS

RNA sequence identity was analyzed with LC-MS following a previously described protocol.[54] Briefly, RNase T1 digests were performed on an automated platform (KingFisher Duo Prime, Thermo Fisher Scientific; BindIt software v4.0) using RNase T1 immobilized on magnetic beads. A 96-deep-well plate was prepared with 50 μL SMART Digest buffer containing RNA (row A) and 50 μL SMART Digest buffer with RNase-coated beads (row G). The instrument

transferred beads to row A and digested at 37 °C for 10 min (eGFP), with bead resuspension maintained by repeated magnetic-comb insertion at "Fast" mixing speed. Digests were analyzed by IP-RP HPLC-MS on a Vanquish UHPLC coupled to an Orbitrap Exploris 240 (Thermo Fisher Scientific) with a DNAPac RP column (2.1 mm I.D.). Chromatographic and MS parameters followed the referenced protocol unless noted earlier.

Quantification of dsRNA with Lumit bioluminescent immunoassay

dsRNA impurities were quantified using the Lumit dsRNA Detection Assay (Promega, Cat. W2041), according to the manufacturer's instructions with minor modifications. The assay is based on NanoLuc Binary Technology (NanoBiT), in which dsRNA-binding domains fused to complementary luciferase fragments (LgBiT and SmBiT) reconstitute an active enzyme upon dsRNA binding, generating luminescence proportional to dsRNA concentration. The assay buffer was freshly prepared before each run. A dsRNA standard curve (0–2.5 ng mL^{-1}) was generated by 2-fold serial dilution in assay buffer and included in triplicate on each plate. DNase-treated, oligo-dT-purified mRNA samples were diluted to 20–200 ng mL^{-1} total RNA (or 200–2,000 ng mL^{-1} for NaCl-buffer samples). Fifty microliters of each standard or sample were dispensed into black 96-well plates, followed by 50 µL of freshly prepared sensor reagent (Lumit dsRNA Sensor-SmBiT and Sensor-LgBiT diluted in assay buffer). Plates were shaken at 300 rpm for 1 min, incubated at 21°C for 60 min, and then supplemented with 25 µL of Lumit Detection Substrate per well. Luminescence was recorded after 15 min using a plate reader in luminescence detection mode. Raw relative light unit (RLU) values were background-corrected using the 0 ng mL^{-1} standard. Standard curves were fitted in GraphPad Prism using linear regression (log-log scaling for visualization), and dsRNA concentrations were interpolated and adjusted for sample dilution. Results were reported both as % dsRNA relative to total RNA mass and as pg dsRNA per µg mRNA.

Protein and cytokine expression analysis

Cell culture

THP-1 cells were cultured as a suspension. Cells were seeded at a density of 0.2×10^6 cells/mL in RPMI-1640 medium supplemented with 10% (v/v) heat-inactivated fetal calf serum (FCS), 2 mM L-glutamine, 100 IU/mL penicillin, and 100 µg/mL streptomycin and incubated at 37°C in a humidified atmosphere of 5% CO_2. Cell medium was replaced, and cell density was maintained every 2–3 days to achieve optimal growth.

Cell culture, mRNA transfection, and flow cytometric analysis

For transfection experiments, THP-1 cells were seeded at 0.5×10^6 cells per mL (1 mL per well) in 12-well plates (Corning Costar, USA). TransIT-mRNA Transfection Reagent (Mirus Bio, USA) was used according to the manufacturer's protocol. Briefly, 1 µg of eGFP mRNA was diluted in 100 µL Opti-MEM (Thermo Fisher Scientific, USA), mixed with 2 µL TransIT-mRNA Reagent and 2 µL TransIT-mRNA Boost Reagent. After a 5-min incubation at room temperature, the transfection mix was added dropwise to the cells, which were then incubated for 24 h at 37°C, 5% CO_2. After incubation, each cell suspension was transferred to a 1.5 mL microcentrifuge tube and centrifuged at 5,000 × g for 5 min. The cell culture supernatant was collected and stored at −20°C for subsequent cytokine bead array (CBA) and IFN-β ELISA analyses. Cell pellets were resuspended in 0.5 mL of 0.1% bovine serum albumin (BSA) in phosphate-buffered saline (PBS) and stained with 0.5 µL LIVE/DEAD Fixable Violet Dead Cell Stain (Thermo Fisher Scientific, USA) for 20 min at 4°C, protected from light. Staining was stopped by the addition of 1 mL 0.1% BSA in PBS, followed by centrifugation at 5,000 × g for 5 min. Pellets were resuspended in 200 µL of 2% paraformaldehyde (PFA) in PBS to fix the cells, transferred to a 96-well plate, and stored at 4°C until flow cytometric analysis. Transfection efficiency was quantified by assessing intracellular eGFP (excitation at 488 nm, emission peak at 511 nm) using a Cytek Aurora 5-laser spectral flow cytometer (Cytek Biosciences, California, USA) and analyzed with FCS Express software (De Novo Software, California, USA). Only events corresponding to live cells (i.e., cells that were not stained violet with the viability LIVE/DEAD dye) were included for subsequent analysis of eGFP-positive cells for transfection efficiency calculations. All settings and gating strategies were standardized across experiments.

Cytokine release analysis

THP-1 cell-conditioned medium was analyzed for cytokine release using Cytometric Bead Array (CBA) (BD Biosciences, USA). The following cytokines were assessed: IFN-α (catalog no. 560379), IL-1β (catalog no. 558279), CCL5 (catalog no. 558324), CCL3 (catalog no. 558325), CXCL10 (catalog no. 558280), and CXCL8 (catalog no. 558277). Reagents and buffers were prepared using the BD CBA Human Soluble Protein Master Buffer Kit (catalog no. 558265) according to the manufacturer's instructions. The concentration of IFN-β in conditioned media was evaluated by ELISA (R&D Systems, Minnesota, USA; catalog no. DY814-05). Spectrophotometric readings were taken at 450 nm with a 570 nm correction filter using a Tecan Infinite M200 plate reader coupled with Magellan software.

Statistical analysis, reproducibility, and data visualization

Unless stated otherwise, all results are reported as mean ± SD from $n = 3$ independent IVT-oligo-dT recycling experiments, each comprising five sequential IVT-oligo-dT cycles. Analytical assays (LC-MS, CGE, AEX-HPLC, dsRNA quantification, and cytokine measurements) were performed on at least three independent samples, yielding consistent results. No data were excluded. Statistical analyses combined parametric and nonparametric approaches to summarize central tendency, assess cycle-to-cycle stability, and compare cumulative yields between conditions. Welch's t tests were used to compare mean yields between buffers, with 95% confidence intervals reported for the difference in means (Δ). Kruskal-Wallis tests were used to assess cycle effects within each buffer (e.g., yield, capping, dsRNA, and eGFP expression). Because the five sequential cycles of each experiment are combined to represent the cumulative five-cycle product, quality attributes of

the pooled product (5′ capping efficiency, dsRNA content, and eGFP expression) were calculated for each campaign as the yield-weighted mean across cycles and compared with the standard process by two-sided Welch's t tests, with 95% confidence intervals reported for the difference in means (Δ). The independent experimental unit for these comparisons is the campaign (n = 3), not the individual cycle. For post-hoc cycle-pair comparisons, Benjamini-Hochberg adjustment was applied separately within each buffer-endpoint family. Cytokine measurements for the NaCl- and CH_3COOK-derived sample sets were acquired separately, and $\log_2$ fold changes for each sample set were calculated relative to the arithmetic mean of the standard-process measurements associated with it. Because each fold change is referenced to an estimated control mean specific to its sample set, the cytokine comparisons are reported descriptively based on directional reproducibility across the three independent experiments, and no significance testing was applied.

Data processing and figure generation were performed in R (version 4.5.0; R Core Team, Vienna, Austria) using the packages tidyverse, ggplot2, dplyr, cowplot, patchwork, reshape2, readxl, openxlsx, shiny, DT, MSnbase, xcms, mzR, ggpubr, pheatmap, and rstatix, together with base stats, stringr, and tidyr for data manipulation and statistical testing. Figures were rendered in ggplot2 and assembled with cowplot and patchwork.

DATA AND CODE AVAILABILITY

All data supporting the findings of this study are provided in the article, supplemental information, and Table S5. Analysis scripts used for KPI and CQA visualization and statistical testing are available at https://github.com/Adnair-94/RNA-CQA-Analysis.

ACKNOWLEDGMENTS

The authors thank Dr. Melanie Lee (Nanoclustering) for designing the graphical abstract. Figures in the main text were created by the authors using BioRender.com and R. The authors also thank Dr. Emma N. Welbourne and James Grinham for their support with preliminary data acquisition and analysis. The author(s) declare that financial support was received for the research, authorship, and/or publication of this article. This work was supported through the UK-Southeast Asia Vaccine Manufacturing Research Hub. The Hub is funded by the Department of Health and Social Care using the UK Government's International Development program (formerly "UK aid") and is managed by the Engineering and Physical Sciences Research Council. The views expressed in this publication are those of the author(s) and not necessarily those of the Department of Health and Social Care. K.M.S. is funded by a UKRI Engineering Biology Mission Award (BB/Y007514/1) awarded to Z.K., H.E.C., M.J.D., and C.M. The study was co-funded by the Wellcome Leap R3 Program. The funders had no role in study design, data collection, analysis, interpretation, or manuscript preparation. Open-access publication was supported by the University of Sheffield Institutional Open Access Fund. For the purpose of open access, the author has applied a Creative Commons Attribution (CC BY) licence to any Author-Accepted Manuscript version arising.

AUTHOR CONTRIBUTIONS

A.N. and Z.K. conceptualized the study. A.N., J.Q., M.M., and Z.K. designed the experiments. J.Q. and M.P. developed the oligo-dT methodology. A.N., J.Q., M.P., T.C.M., C.A.E., K.A.L., and Z.V.R.A. conducted the investigation. T.C.M., C.A.E., A.N., Z.V.R.A., and M.J.D. performed analytical work and interpreted the data. K.M.S., H.E.C., and C.M. conducted the cell-based assays. A.N. and Z.K. prepared the original draft and visualizations. M.J.D. and Z.K. supervised the work. Z.K. acquired funding. All authors reviewed and edited the manuscript.

DECLARATION OF INTERESTS

A patent application related to the integrated IVT-oligo-dT recycling process (WO 2025/109302) has been filed by the authors and their institution. A.N., C.A.E., and M.M. are shareholders of RNA Forge, Ltd. (UK company number 16612680). Z.K. and M.J.D. are co-founders, board members, and shareholders of RNA Forge, Ltd. All other authors declare no competing interests.

DECLARATION OF GENERATIVE AI AND AI-ASSISTED TECHNOLOGIES IN THE WRITING PROCESS

During the preparation of this work, the authors used ChatGPT (OpenAI) for language refinement, structural refinement, and consistency checks, and Grammarly for grammar and style correction. After using these tools, the authors reviewed and edited the content as needed and take full responsibility for the content of the publication.

SUPPLEMENTAL INFORMATION

Supplemental information can be found online at https://doi.org/10.1016/j.omtn.2026.103089.

REFERENCES

1. Qin, S., Tang, X., Chen, Y., Chen, K., Fan, N., Xiao, W., Zheng, Q., Li, G., Teng, Y., Wu, M., and Song, X. (2022). mRNA-based therapeutics: powerful and versatile tools to combat diseases. Signal Transduct. Target. Ther. *7*, 166. https://doi.org/10.1038/s41392-022-01007-w.
2. Damase, T.R., Sukhovershin, R., Boada, C., Taraballi, F., Pettigrew, R.I., and Cooke, J.P. (2021). The limitless future of RNA therapeutics. Front. Bioeng. Biotechnol. *9*, 628137. https://doi.org/10.3389/fbioe.2021.628137.
3. Pardi, N., Hogan, M.J., Porter, F.W., and Weissman, D. (2018). mRNA vaccines—a new era in vaccinology. Nat. Rev. Drug Discov. *17*, 261–279. https://doi.org/10.1038/nrd.2017.243.
4. Kwon, H., Kim, M., Seo, Y., Moon, Y.S., Lee, H.J., Lee, K., and Lee, H. (2018). Emergence of synthetic mRNA: In vitro synthesis of mRNA and its applications in regenerative medicine. Biomaterials *156*, 172–193. https://doi.org/10.1016/j.biomaterials.2017.11.034.
5. Żak, M.M., and Zangi, L. (2025). Clinical development of therapeutic mRNA applications. Mol. Ther. *33*, 2583–2609. https://doi.org/10.1016/j.ymthe.2025.03.034.
6. Whitley, J., Zwolinski, C., Denis, C., Maughan, M., Hayles, L., Clarke, D., Snare, M., Liao, H., Chiou, S., Marmura, T., et al. (2022). Development of mRNA manufacturing for vaccines and therapeutics: mRNA platform requirements and development of a scalable production process to support early phase clinical trials. Transl. Res. *242*, 38–55. https://doi.org/10.1016/j.trsl.2021.11.009.
7. Daniel, S., Kis, Z., Kontoravdi, C., and Shah, N. (2022). Quality by Design for enabling RNA platform production processes. Trends Biotechnol. *40*, 1213–1228. https://doi.org/10.1016/j.tibtech.2022.03.012.
8. Szabó, G.T., Mahiny, A.J., and Vlatkovic, I. (2022). COVID-19 mRNA vaccines: Platforms and current developments. Mol. Ther. *30*, 1850–1868. https://doi.org/10.1016/j.ymthe.2022.02.016.
9. Nair, A., Loveday, K.A., Kenyon, C., Qu, J., and Kis, Z. (2024). Quality by Digital Design for developing platform RNA vaccine and therapeutic manufacturing processes. Methods Mol. Biol. *2786*, 339–364. https://doi.org/10.1007/978-1-0716-3770-8_16.
10. Davidopoulou, C., Kouvelas, D., and Ouranidis, A. (2024). COMPARING vaccine manufacturing technologies recombinant DNA vs in vitro transcribed (IVT) mRNA. Sci. Rep. *14*, 21742. https://doi.org/10.1038/s41598-024-67797-x.
11. Rosa, S.S., Prazeres, D.M.F., Azevedo, A.M., and Marques, M.P.C. (2021). mRNA vaccines manufacturing: Challenges and bottlenecks. Vaccine *39*, 2190–2200. https://doi.org/10.1016/j.vaccine.2021.03.038.
12. Beckert, B., and Masquida, B. (2011). Synthesis of RNA by in vitro transcription. Methods Mol. Biol. *703*, 29–41. https://doi.org/10.1007/978-1-59745-248-9_3.
13. Pardi, N., Muramatsu, H., Weissman, D., and Karikó, K. (2013). In vitro transcription of long RNA containing modified nucleosides. Methods Mol. Biol. *969*, 29–42. https://doi.org/10.1007/978-1-62703-260-5_2.

14. Milligan, J.F., and Uhlenbeck, O.C. (1989). [5] Synthesis of small RNAs using T7 RNA polymerase. Methods Enzymol. *180*, 51–62. https://doi.org/10.1016/0076-6879(89)80091-6.

15. Henderson, J.M., Ujita, A., Hill, E., Yousif-Rosales, S., Smith, C., Ko, N., McReynolds, T., Cabral, C.R., Escamilla-Powers, J.R., and Houston, M.E. (2021). Cap 1 messenger RNA synthesis with co-transcriptional CleanCap® analog by in vitro transcription. Curr. Protoc. *1*, e39. https://doi.org/10.1002/cpz1.39.

16. Zhang, L., More, K.R., Ojha, A., Jackson, C.B., Quinlan, B.D., Li, H., He, W., Farzan, M., Pardi, N., and Choe, H. (2023). Effect of mRNA-LNP components of two globally marketed COVID-19 vaccines on efficacy and stability. NPJ Vaccines *8*, 156. https://doi.org/10.1038/s41541-023-00751-6.

17. Vogel, A.B., Kanevsky, I., Che, Y., Swanson, K.A., Muik, A., Vormehr, M., Kranz, L.M., Walzer, K.C., Hein, S., Güler, A., et al. (2021). BNT162b vaccines protect rhesus macaques from SARS-CoV-2. Nature *592*, 283–289. https://doi.org/10.1038/s41586-021-03275-y.

18. Kis, Z., Tak, K., Ibrahim, D., Papathanasiou, M.M., Chachuat, B., Shah, N., and Kontoravdi, C. (2022). Pandemic-response adenoviral vector and RNA vaccine manufacturing. NPJ Vaccines *7*, 29. https://doi.org/10.1038/s41541-022-00447-3.

19. Kis, Z., Kontoravdi, C., Shattock, R., and Shah, N. (2020). Resources, production scales and time required for producing RNA vaccines for the global pandemic demand. Vaccines (Basel) *9*, 3. https://doi.org/10.3390/vaccines9010003.

20. Pons Royo, M.d.C., Arnold, T., Rodriguez, I.P., Ostrovsky, N., Al-Jazrawe, M., Hatas, A., Myerson, A.S., and Braatz, R.D. (2025). Continuous purification of mRNA by precipitation and sequential TFF. Sep. Purif. Technol. *379*, 134837. https://doi.org/10.1016/j.seppur.2025.134837.

21. Nourafkan, E., Kenyon, C., Nair, A., Loveday, K.A., Welbourne, E.N., Tao, M., Ahmed, M., Middleton, J., Dickman, M.J., Brown, S.F., et al. (2024). An experimental and modeling approach to study tangential flow filtration performance for mRNA drug substance purification. Biotechnol. J. *19*, e202400473. https://doi.org/10.1002/biot.202400473.

22. Javidanbardan, A., Wang, Z., Kostic, A., Behboudi, A., and Zydney, A.L. (2025). Single-pass tangential flow filtration (SPTFF) for continuous mRNA concentration and purification. J. Memb. Sci. *719*, 123730. https://doi.org/10.1016/j.memsci.2025.123730.

23. Baiersdörfer, M., Boros, G., Muramatsu, H., Mahiny, A., Vlatkovic, I., Sahin, U., and Karikó, K. (2019). A facile method for the removal of dsRNA contaminant from in vitro-transcribed mRNA. Mol. Ther. Nucleic Acids *15*, 26–35. https://doi.org/10.1016/j.omtn.2019.02.018.

24. Krušič, A., Mencin, N., Leban, M., Nett, E., Perković, M., Sahin, U., Megušar, P., Štrancar, A., and Sekirnik, R. (2025). Reverse-phase chromatography removes double-stranded RNA, fragments, and residual template to decrease immunogenicity and increase cell potency of mRNA and saRNA. Mol. Ther. Nucleic Acids *36*, 102491. https://doi.org/10.1016/j.omtn.2025.102491.

25. Weissman, D., Pardi, N., Muramatsu, H., and Karikó, K. (2013). HPLC purification of in vitro transcribed long RNA. Methods Mol. Biol. *969*, 43–54. https://doi.org/10.1007/978-1-62703-260-5_3.

26. Clark, N.E., Kozarski, M., Asci, S.D., Van den Heuvel, J., Schraut, M.R., Winters, R.A., Kearns, K., Scanlon, T.C., and Dillen, S. (2025). Removal of dsRNA byproducts using affinity chromatography. Mol. Ther. Nucleic Acids *36*, 102549. https://doi.org/10.1016/j.omtn.2025.102549.

27. Qu, J., Nair, A., Muir, G.W., Loveday, K.A., Yang, Z., Nourafkan, E., Welbourne, E.N., Maamra, M., Dickman, M.J., and Kis, Z. (2024). Quality by design for mRNA platform purification based on continuous oligo-dT chromatography. Mol. Ther. Nucleic Acids *35*, 102333. https://doi.org/10.1016/j.omtn.2024.102333.

28. Mencin, N., Štepec, D., Margon, A., Vidič, J., Dolenc, D., Simčič, T., Rotar, S., Sekirnik, R., Štrancar, A., and Černigoj, U. (2023). Development and scale-up of oligo-dT monolithic chromatographic column for mRNA capture through understanding of base-pairing interactions. Sep. Purif. Technol. *304*, 122320. https://doi.org/10.1016/j.seppur.2022.122320.

29. Korenč, M., Mencin, N., Puc, J., Skok, J., Nemec, K.Š., Celjar, A.M., Gagnon, P., Štrancar, A., and Sekirnik, R. (2021). Chromatographic purification with CIMmultus™ Oligo dT increases mRNA stability. Cell Gene Ther. Insights *7*, 1207–1216. https://doi.org/10.18609/cgti.2021.161.

30. Dewar, E.A., Guterstam, P., Holland, D., Lindman, S., Lundbäck, P., Brito dos Santos, S., Wang, S.-C., and Swartz, A.R. (2024). Improved mRNA affinity chromatography binding capacity and throughput using an oligo-dT immobilized electrospun polymer nanofiber adsorbent. J. Chromatogr. A *1717*, 464670. https://doi.org/10.1016/j.chroma.2024.464670.

31. Cavac, E., Ramírez-Tapia, L.E., and Martin, C.T. (2021). High-salt transcription of DNA cotethered with T7 RNA polymerase to beads generates increased yields of highly pure RNA. J. Biol. Chem. *297*, 100999. https://doi.org/10.1016/j.jbc.2021.100999.

32. Milligan, J.F., Groebe, D.R., Witherell, G.W., and Uhlenbeck, O.C. (1987). Oligoribonucleotide synthesis using T7 RNA polymerase and synthetic DNA templates. Nucleic Acids Res. *15*, 8783–8798. https://doi.org/10.1093/nar/15.21.8783.

33. Rosa, S.S., Nunes, D., Antunes, L., Prazeres, D.M.F., Marques, M.P.C., and Azevedo, A.M. (2022). Maximizing mRNA vaccine production with Bayesian optimization. Biotechnol. Bioeng. *119*, 3127–3139. https://doi.org/10.1002/bit.28216.

34. Skok, J., Megušar, P., Vodopivec, T., Pregeljc, D., Mencin, N., Korenč, M., Krušič, A., Celjar, A.M., Pavlin, N., Krušič, J., et al. (2022). Gram-scale mRNA production using a 250-mL single-use bioreactor. Chem. Ing. Tech. *94*, 1928–1935. https://doi.org/10.1002/cite.202200133.

35. Guo, L., Liu, Z., Song, S., Yao, W., Yang, M., and Chen, G. (2024). Maximizing the mRNA productivity for in vitro transcription by optimization of fed-batch strategy. Biochem. Eng. J. *210*, 109412. https://doi.org/10.1016/j.bej.2024.109412.

36. Boman, J., Marušič, T., Seravalli, T.V., Skok, J., Pettersson, F., Nemec, K.Š., Widmark, H., and Sekirnik, R. (2024). Quality by design approach to improve quality and decrease cost of in vitro transcription of mRNA using design of experiments. Biotechnol. Bioeng. *121*, 3415–3427. https://doi.org/10.1002/bit.28806.

37. Dousis, A., Ravichandran, K., Hobert, E.M., Moore, M.J., and Rabideau, A.E. (2023). An engineered T7 RNA polymerase that produces mRNA free of immunostimulatory byproducts. Nat. Biotechnol. *41*, 560–568. https://doi.org/10.1038/s41587-022-01525-6.

38. Zhu, B., Tabor, S., and Richardson, C.C. (2014). Syn5 RNA polymerase synthesizes precise run-off RNA products. Nucleic Acids Res. *42*, e33. https://doi.org/10.1093/nar/gkt1193.

39. Miller, M., Alvizo, O., Baskerville, S., Chintala, A., Chng, C., Dassie, J., Dorigatti, J., Huisman, G., Jenne, S., Kadam, S., et al. (2024). An engineered T7 RNA polymerase for efficient co-transcriptional capping with reduced dsRNA byproducts in mRNA synthesis. Faraday Discuss. *252*, 431–449. https://doi.org/10.1039/d4fd00023d.

40. Nair, A., and Kis, Z. (2024). Bacteriophage RNA polymerases: catalysts for mRNA vaccines and therapeutics. Front. Mol. Biosci. *11*, 1504876. https://doi.org/10.3389/fmolb.2024.1504876.

41. Chan, S.H., Tirole, L., Kneller, D., Kelley, T.M., Dai, N., and Robb, G.B. (2023). Co-transcriptional capping using an RNA capping enzyme-T7 RNA polymerase fusion protein. Preprint at bioRxiv. https://doi.org/10.1101/2023.10.28.564488.

42. Nwokeoji, A.O., Chou, T., and Nwokeoji, E.A. (2023). Low resource integrated platform for production and analysis of capped mRNA. ACS Synth. Biol. *12*, 329–339. https://doi.org/10.1021/acssynbio.2c00609.

43. Malagoda Pathiranage, K., and Martin, C.T. (2023). A simple approach to improving RNA synthesis: Salt inhibition of RNA rebinding coupled with strengthening promoter binding by a targeted gap in the DNA. Methods Enzymol. *691*, 209–222. https://doi.org/10.1016/bs.mie.2023.06.001.

44. Lenk, R., Kleindienst, W., Szabó, G.T., Baiersdörfer, M., Boros, G., Keller, J.M., Mahiny, A.J., and Vlatkovic, I. (2024). Understanding the impact of in vitro transcription byproducts and contaminants. Front. Mol. Biosci. *11*, 1426129. https://doi.org/10.3389/fmolb.2024.1426129.

45. Gholamalipour, Y., Johnson, W.C., and Martin, C.T. (2019). Efficient inhibition of RNA self-primed extension by addition of competing 3′-capture DNA-improved RNA synthesis by T7 RNA polymerase. Nucleic Acids Res. *47*, e118. https://doi.org/10.1093/nar/gkz700.

46. Piao, X., Yadav, V., Wang, E., Chang, W., Tau, L., Lindenmuth, B.E., and Wang, S.X. (2022). Double-stranded RNA reduction by chaotropic agents during in vitro

transcription of messenger RNA. Mol. Ther. Nucleic Acids *29*, 618–624. https://doi.org/10.1016/j.omtn.2022.08.001.

47. Maslak, M., and Martin, C.T. (1994). Effects of solution conditions on the steady-state kinetics of initiation of transcription by T7 RNA polymerase. Biochemistry *33*, 6918–6924. https://doi.org/10.1021/bi00188a022.

48. MalagodaPathiranage, K., Cavac, E., Chen, T.-H., Roy, B., and Martin, C.T. (2023). High-salt transcription from enzymatically gapped promoters nets higher yields and purity of transcribed RNAs. Nucleic Acids Res. *51*, e36. https://doi.org/10.1093/nar/gkad027.

49. Liao, J.C., and Tung, Y.-C. (2023). Device for continuously producing and analyzing RNA (U.S. Patent Application Publication US20230028771A1).

50. Akama, S., Yamamura, M., and Kigawa, T. (2012). A multiphysics model of in vitro transcription coupling enzymatic reaction and precipitation formation. Biophys. J. *102*, 221–230. https://doi.org/10.1016/j.bpj.2011.12.014.

51. Marušič, T., Faganel, T., and Sekirnik, R. (2026). High-throughput monitoring of free Mg2+ in IVT reaction reveals effects of free Mg2+ on mRNA synthesis and DNA digestion. Anal. Bioanal. Chem. *418*, 3983–3997. https://doi.org/10.1007/s00216-026-06542-x.

52. Shieh, Y., Swartz, A.R., and Rustandi, R.R. (2024). Detection of residual T7 RNA polymerase used in mRNA in vitro transcription by Simple Western. Electrophoresis *45*, 1834–1839. https://doi.org/10.1002/elps.202400044.

53. Welbourne, E.N., Loveday, K.A., Nair, A., Nourafkan, E., Qu, J., Cook, K., Kis, Z., and Dickman, M.J. (2024). Anion exchange HPLC monitoring of mRNA in vitro transcription reactions to support mRNA manufacturing process development. Front. Mol. Biosci. *11*, 1250833. https://doi.org/10.3389/fmolb.2024.1250833.

54. Welbourne, E.N., Copley, R.J., Owen, G.R., Evans, C.A., Isoko, K., Cook, K., Cordiner, J., Kis, Z., Moghadam, P.Z., and Dickman, M.J. (2025). Mass spectrometry-based mRNA sequence mapping via complementary RNase digests and bespoke visualisation tools. Analyst *150*, 1012–1021. https://doi.org/10.1039/d5an00033e.

OMTN, Volume *37*

Supplemental information

Integrated *in vitro* transcription and oligo-dT affinity chromatography enable multi-cycle reagent recycling for mRNA manufacturing

Adithya Nair, Jixin Qu, Manoj Pohare, Thomas C. Minshull, Caroline A. Evans, Klaudia M. Slowik, Kate A. Loveday, Zoe V.R. Ashcroft, Mabrouka Maamra, Helen E. Colley, Craig Murdoch, Mark J. Dickman, and Zoltán Kis

Template DNA Sequences

Enhanced green fluorescent protein (eGFP) sequence

AGGAAATAAGAGAGAAAAGAAGAGTAAGAAGAAATATAAGAGCCACCATGGTGAGCAAGGGCGA
GGAGCTGTTCACCGGGGTGGTGCCCATCCTGGTCGAGCTGGACGGCGACGTAAACGGCCACAA
GTTCAGCGTGTCCGGCGAGGGCGAGGGCGATGCCACCTACGGCAAGCTGACCCTGAAGTTCAT
CTGCACCACCGGCAAGCTGCCCGTGCCCTGGCCCACCCTCGTGACCACCCTGACCTACGGCGT
GCAGTGCTTCAGCCGCTACCCCGACCACATGAAGCAGCACGACTTCTTCAAGTCCGCCATGCCC
GAAGGCTACGTCCAGGAGCGCACCATCTTCTTCAAGGACGACGGCAACTACAAGACCCGCGCC
GAGGTGAAGTTCGAGGGCGACACCCTGGTGAACCGCATCGAGCTGAAGGGCATCGACTTCAAG
GAGGACGGCAACATCCTGGGGCACAAGCTGGAGTACAACTACAACAGCCACAACGTCTATATCA
TGGCCGACAAGCAGAAGAACGGCATCAAGGTGAACTTCAAGATCCGCCACAACATCGAGGACG
GCAGCGTGCAGCTCGCCGACCACTACCAGCAGAACACCCCCATCGGCGACGGCCCCGTGCTGC
TGCCCGACAACCACTACCTGAGCACCCAGTCCGCCCTGAGCAAAGACCCCAACGAGAAGCGCG
ATCACATGGTCCTGCTGGAGTTCGTGACCGCCGCCGGGATCACTCTCGGCATGGACGAGCTGT
ACAAGTAAGCGGCCGCTTAATTAAGCTGCCTTCTGCGGGGCTTGCCTTCTGGCCATGCCCTTCT
TCTCTCCCTTGCACCTGTACCTCTTGGTCTTTGAATAAAGCCTGAGTAGGAAGCGTACGAAAAAA
AAAAAAAAAAAAAAAAAAAAAAAAAAAAAAAAAAAAAAAAAAAA

SARS-CoV-2 Spike protein (CSP) sequence

AGAATAAACTAGTATTCTTCTGGTCCCCACAGACTCAGAGAGAACCCGCCACCATGTTCGTGTTC
CTGGTGCTGCTGCCTCTGGTGTCCAGCCAGTGTGTGAACCTGACCACCAGAACACAGCTGCCTC
CAGCCTACACCAACAGCTTTACCAGAGGCGTGTACTACCCCGACAAGGTGTTCAGATCCAGCGT
GCTGCACTCTACCCAGGACCTGTTCCTGCCTTTCTTCAGCAACGTGACCTGGTTCCACGCCATC
CACGTGTCCGGCACCAATGGCACCAAGAGATTCGACAACCCCGTGCTGCCCTTCAACGACGGG
GTGTACTTTGCCAGCACCGAGAAGTCCAACATCATCAGAGGCTGGATCTTCGGCACCACACTGG
ACAGCAAGACCCAGAGCCTGCTGATCGTGAACAACGCCACCAACGTGGTCATCAAAGTGTGCGA
GTTCCAGTTCTGCAACGACCCCTTCCTGGGCGTCTACTACCACAAGAACAACAAGAGCTGGATG
GAAAGCGAGTTCCGGGTGTACAGCAGCGCCAACAACTGCACCTTCGAGTACGTGTCCCAGCCTT
TCCTGATGGACCTGGAAGGCAAGCAGGGCAACTTCAAGAACCTGCGCGAGTTCGTGTTTAAGAA
CATCGACGGCTACTTCAAGATCTACAGCAAGCACACCCCTATCAACCTCGTGCGGGATCTGCCT
CAGGGCTTCTCTGCTCTGGAACCCCTGGTGGATCTGCCCATCGGCATCAACATCACCCGGTTTC
AGACACTGCTGGCCCTGCACAGAAGCTACCTGACACCTGGCGATAGCAGCAGCGGATGGACAG
CTGGTGCCGCCGCTTACTATGTGGGCTACCTGCAGCCTAGAACCTTCCTGCTGAAGTACAACGA
GAACGGCACCATCACCGACGCCGTGGATTGTGCTCTGGATCCTCTGAGCGAGACAAAGTGCAC
CCTGAAGTCCTTCACCGTGGAAAAGGGCATCTACCAGACCAGCAACTTCCGGGTGCAGCCCACC
GAATCCATCGTGCGGTTCCCCAATATCACCAATCTGTGCCCCTTCGGCGAGGTGTTCAATGCCA
CCAGATTCGCCTCTGTGTACGCCTGGAACCGGAAGCGGATCAGCAATTGCGTGGCCGACTACTC
CGTGCTGTACAACTCCGCCAGCTTCAGCACCTTCAAGTGCTACGGCGTGTCCCCTACCAAGCTG
AACGACCTGTGCTTCACAAACGTGTACGCCGACAGCTTCGTGATCCGGGGAGATGAAGTGCGG
CAGATTGCCCCTGGACAGACAGGCAAGATCGCCGACTACAACTACAAGCTGCCCGACGACTTCA
CCGGCTGTGTGATTGCCTGGAACAGCAACAACCTGGACTCCAAAGTCGGCGGCAACTACAATTA
CCTGTACCGGCTGTTCCGGAAGTCCAATCTGAAGCCCTTCGAGCGGGACATCTCCACCGAGATC
TATCAGGCCGGCAGCACCCCTTGTAACGGCGTGGAAGGCTTCAACTGCTACTTCCCACTGCAGT
CCTACGGCTTTCAGCCCACAAATGGCGTGGGCTATCAGCCCTACAGAGTGGTGGTGCTGAGCTT
CGAACTGCTGCATGCCCCTGCCACAGTGTGCGGCCCTAAGAAAAGCACCAATCTCGTGAAGAAC
AAATGCGTGAACTTCAACTTCAACGGCCTGACCGGCACCGGCGTGCTGACAGAGAGCAACAAGA
AGTTCCTGCCATTCCAGCAGTTTGGCCGGGATATCGCCGATACCACAGACGCCGTTAGAGATCC
CCAGACACTGGAAATCCTGGACATCACCCCTTGCAGCTTCGGCGGAGTGTCTGTGATCACCCCT
GGCACCAACACCAGCAATCAGGTGGCAGTGCTGTACCAGGACGTGAACTGTACCGAAGTGCCC

GTGGCCATTCACGCCGATCAGCTGACACCTACATGGCGGGTGTACTCCACCGGCAGCAATGTGT
TTCAGACCAGAGCCGGCTGTCTGATCGGAGCCGAGCACGTGAACAATAGCTACGAGTGCGACAT
CCCCATCGGCGCTGGAATCTGCGCCAGCTACCAGACACAGACAAACAGCCCTCGGAGAGCCAG
AAGCGTGGCCAGCCAGAGCATCATTGCCTACACAATGTCTCTGGGCGCCGAGAACAGCGTGGC
CTACTCCAACAACTCTATCGCTATCCCCACCAACTTCACCATCAGCGTGACCACAGAGATCCTGC
CTGTGTCCATGACCAAGACCAGCGTGGACTGCACCATGTACATCTGCGGCGATTCCACCGAGTG
CTCCAACCTGCTGCTGCAGTACGGCAGCTTCTGCACCCAGCTGAATAGAGCCCTGACAGGGATC
GCCGTGGAACAGGACAAGAACACCCAAGAGGTGTTCGCCCAAGTGAAGCAGATCTACAAGACC
CCTCCTATCAAGGACTTCGGCGGCTTCAATTTCAGCCAGATTCTGCCCGATCCTAGCAAGCCCA
GCAAGCGGAGCTTCATCGAGGACCTGCTGTTCAACAAAGTGACACTGGCCGACGCCGGCTTCAT
CAAGCAGTATGGCGATTGTCTGGGCGACATTGCCGCCAGGGATCTGATTTGCGCCCAGAAGTTT
AACGGACTGACAGTGCTGCCTCCTCTGCTGACCGATGAGATGATCGCCCAGTACACATCTGCCC
TGCTGGCCGGCACAATCACAAGCGGCTGGACATTTGGAGCAGGCGCCGCTCTGCAGATCCCCT
TTGCTATGCAGATGGCCTACCGGTTCAACGGCATCGGAGTGACCCAGAATGTGCTGTACGAGAA
CCAGAAGCTGATCGCCAACCAGTTCAACAGCGCCATCGGCAAGATCCAGGACAGCCTGAGCAG
CACAGCAAGCGCCCTGGGAAAGCTGCAGGACGTGGTCAACCAGAATGCCCAGGCACTGAACAC
CCTGGTCAAGCAGCTGTCCTCCAACTTCGGCGCCATCAGCTCTGTGCTGAACGATATCCTGAGC
AGACTGGACCCTCCTGAGGCCGAGGTGCAGATCGACAGACTGATCACAGGCAGACTGCAGAGC
CTCCAGACATACGTGACCCAGCAGCTGATCAGAGCCGCCGAGATTAGAGCCTCTGCCAATCTGG
CCGCCACCAAGATGTCTGAGTGTGTGCTGGGCCAGAGCAAGAGAGTGGACTTTTGCGGCAAGG
GCTACCACCTGATGAGCTTCCCTCAGTCTGCCCCTCACGGCGTGGTGTTTCTGCACGTGACATA
TGTGCCCGCTCAAGAGAAGAATTTCACCACCGCTCCAGCCATCTGCCACGACGGCAAAGCCCAC
TTTCCTAGAGAAGGCGTGTTCGTGTCCAACGGCACCCATTGGTTCGTGACACAGCGGAACTTCT
ACGAGCCCCAGATCATCACCACCGACAACACCTTCGTGTCTGGCAACTGCGACGTCGTGATCGG
CATTGTGAACAATACCGTGTACGACCCTCTGCAGCCCGAGCTGGACAGCTTCAAAGAGGAACTG
GACAAGTACTTTAAGAACCACACAAGCCCCGACGTGGACCTGGGCGATATCAGCGGAATCAATG
CCAGCGTCGTGAACATCCAGAAAGAGATCGACCGGCTGAACGAGGTGGCCAAGAATCTGAACG
AGAGCCTGATCGACCTGCAAGAACTGGGGAAGTACGAGCAGTACATCAAGTGGCCCTGGTACAT
CTGGCTGGGCTTTATCGCCGGACTGATTGCCATCGTGATGGTCACAATCATGCTGTGTTGCATG
ACCAGCTGCTGTAGCTGCCTGAAGGGCTGTTGTAGCTGTGGCAGCTGCTGCAAGTTCGACGAG
GACGATTCTGAGCCCGTGCTGAAGGGCGTGAAACTGCACTACACATGATGACTCGAGCTGGTAC
TGCATGCACGCAATGCTAGCTGCCCCTTTCCCGTCCTGGGTACCCCGAGTCTCCCCCGACCTCG
GGTCCCAGGTATGCTCCCACCTCCACCTGCCCCACTCACCACCTCTGCTAGTTCCAGACACCTC
CCAAGCACGCAGCAATGCAGCTCAAAACGCTTAGCCTAGCCACACCCCCACGGGAAACAGCAGT
GATTAACCTTTAGCAATAAACGAAAGTTTAACTAAGCTATACTAACCCCAGGGTTGGTCAATTTCG
TGCCAGCCACACCCTGGAGCTAGCAAAAAAAAAAAAAAAAAAAAAAAAAAAAAGCATATGACTA
AAAAAAAAAAAAAAAAAAAAAAAAAAAAAAAAAAAAAAAAAAAAAAAAAAAAAAAAAAAAAAAA
AA

Table S1. Initial reaction compositions for standard IVT and cycle 1 of the IVT-oligo-dT workflows. Stock concentrations are listed alongside the nominal starting concentrations used in each condition.

Component	Stock concentration	Standard IVT	IVT-Oligo-dT
Tris-HCl	1000 mM	40 mM	40 mM
DTT	1000 mM	10 mM	10 mM
Spermidine	6400 mM	2 mM	2 mM
Triton X-100	100% (v/v)	0.01% (v/v)	0.01% (v/v)
[a] NaCl/CH_3COOK	5000/1000 mM	-	100 mM
GuHCl	8000 mM	-	50 mM
$MgCl_2$	1000 mM	42 mM	16 mM
NTP (total)	100 mM	40 mM	10 mM
CleanCap AG	100 mM	10 mM	2.5 mM
T7 RNAP	0.010 mM	0.001 mM	0.00075 mM
Template DNA	0.00050 mM	0.000025 mM	0.000025 mM
Inorganic pyrophosphatase	0.0618 mM	0.0030 mM	0.0030 mM
RNase inhibitor	0.0075 mM	0.00019 mM	0.00019 mM
RNase-free water	-	-	-

[a]*NaCl/CH_3COOK indicates that either NaCl or CH_3COOK was used at 100 mM. Unless otherwise stated, all other component concentrations in the IVT-oligo-dT condition are identical in both buffer systems.*

Table S2. Fixed supplementation before each recycled IVT cycle (cycles 2–5).

Component	Supplemented amount per cycle
$MgCl_2$	80 µmol/cycle
NTP (total)	80 µmol/cycle (20 µmol of each NTP)
Template DNA	200 µg/cycle
All other components	-

Note: Supplementation amounts were fixed charges added before each of cycles 2-5. The 80 µmol $MgCl_2$ charge corresponds to a nominal 10 mM increment in an 8 mL reaction. Residual Mg^{2+} and NTPs from the preceding cycle were not quantified or subtracted; therefore, these values do not represent actual cycle-start concentrations. A dash indicates no between-cycle supplementation.

Table S3. Buffer compositions used for standard oligo-dT and integrated IVT-oligo-dT purification methods.

	**Component**	**Standard oligo-dT**	**IVT-Oligo-dT NaCl buffer**	**IVT-Oligo-dT CH_3COOK buffer**
Equilibration	NaCl	250 mM	100 mM	-
	Phosphate buffer	50 mM	-	-
	EDTA	2 mM	-	-
	Gu-HCl	-	50 mM	50 mM
	Tris-HCl (pH 7.0)	-	40 mM	40 mM
	CH_3COOK	-	-	100 mM
Load	Same as equilibration			
Wash	Same as equilibration			
Elution	Nuclease-free water			
CIP	NaOH		100 mM	

Table S4. Cap analog concentration in flowthrough and residual NTP in elution.

Cycle	Cap analog in flowthrough (NaCl buffer, % relative to cycle 1)	Cap analog in flowthrough (CH_3COOK buffer, % relative to cycle 1)	Residual rNTP in elution (NaCl buffer, %)	Residual rNTP in elution (CH_3COOK buffer, %)
1	100.0	100.0	Not detected	0.4
2	75.9	70.7	Not detected	0.8
3	75.4	60.3	Not detected	0.5
4	54.4	50.9	Not detected	0.3
5	36.9	39.4	Not detected	0.2

Table S5. Source data supporting the main figures. The underlying numerical values used for the quantitative analyses are provided as a separate Excel workbook.

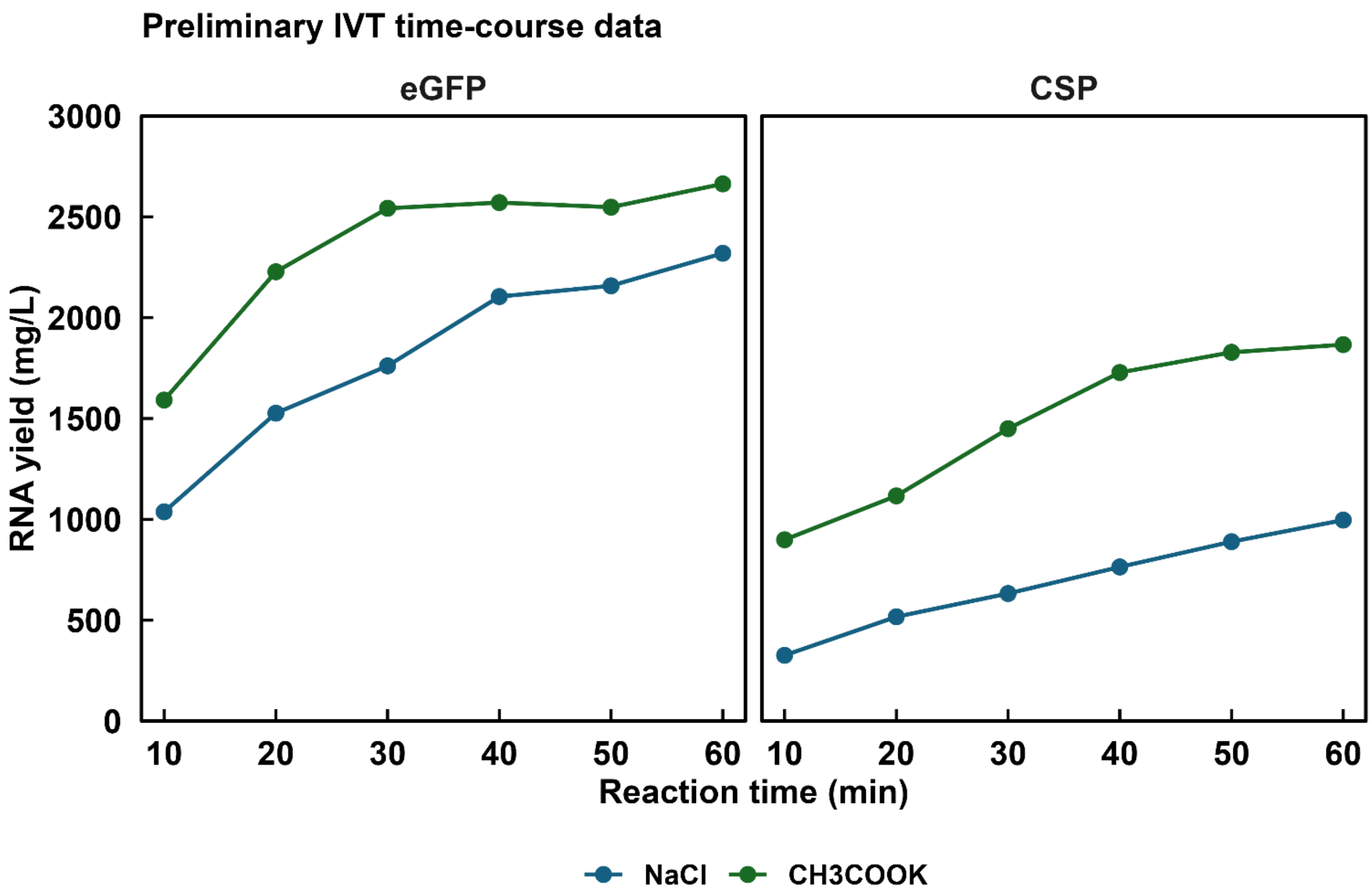

Figure S1. IVT time-course screening under shared-buffer conditions. RNA yield was measured over time for eGFP and CSP transcripts in NaCl- and CH_3COOK-based shared buffers containing 50 mM Gu-HCl. The data were used as preliminary process-development screening to support the buffer-dependent incubation-time rationale in the integrated IVT-oligo-dT recycling workflow. CH_3COOK showed faster apparent RNA accumulation than NaCl under the tested conditions. Data are preliminary screening measurements and were not used for statistical comparison.

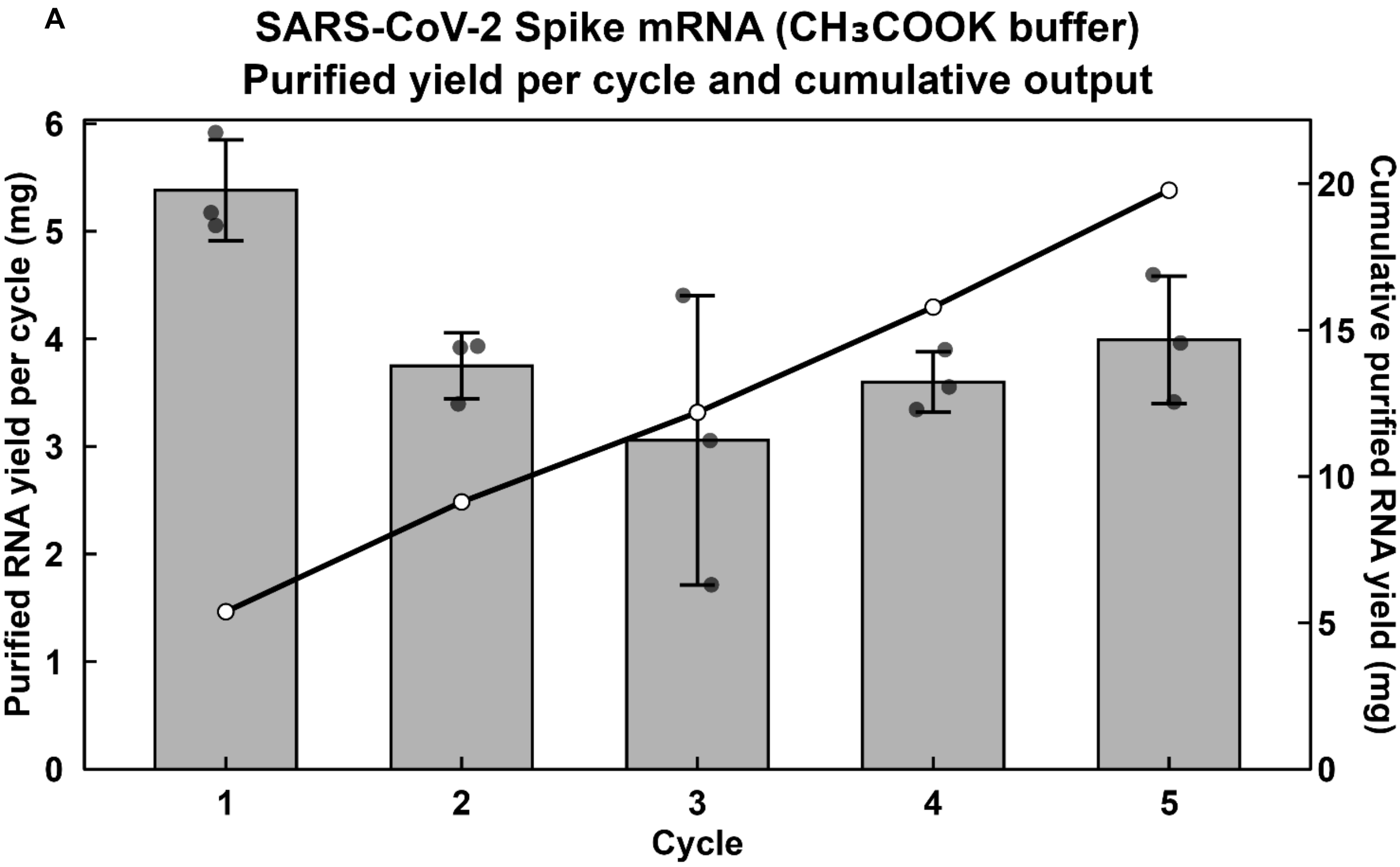
A
SARS-CoV-2 Spike mRNA (CH₃COOK buffer)
Purified yield per cycle and cumulative output
Purified RNA yield per cycle (mg)
Cumulative purified RNA yield (mg)
Cycle
0
1
2
3
4
5
6
0
5
10
15
20
1
2
3
4
5

B

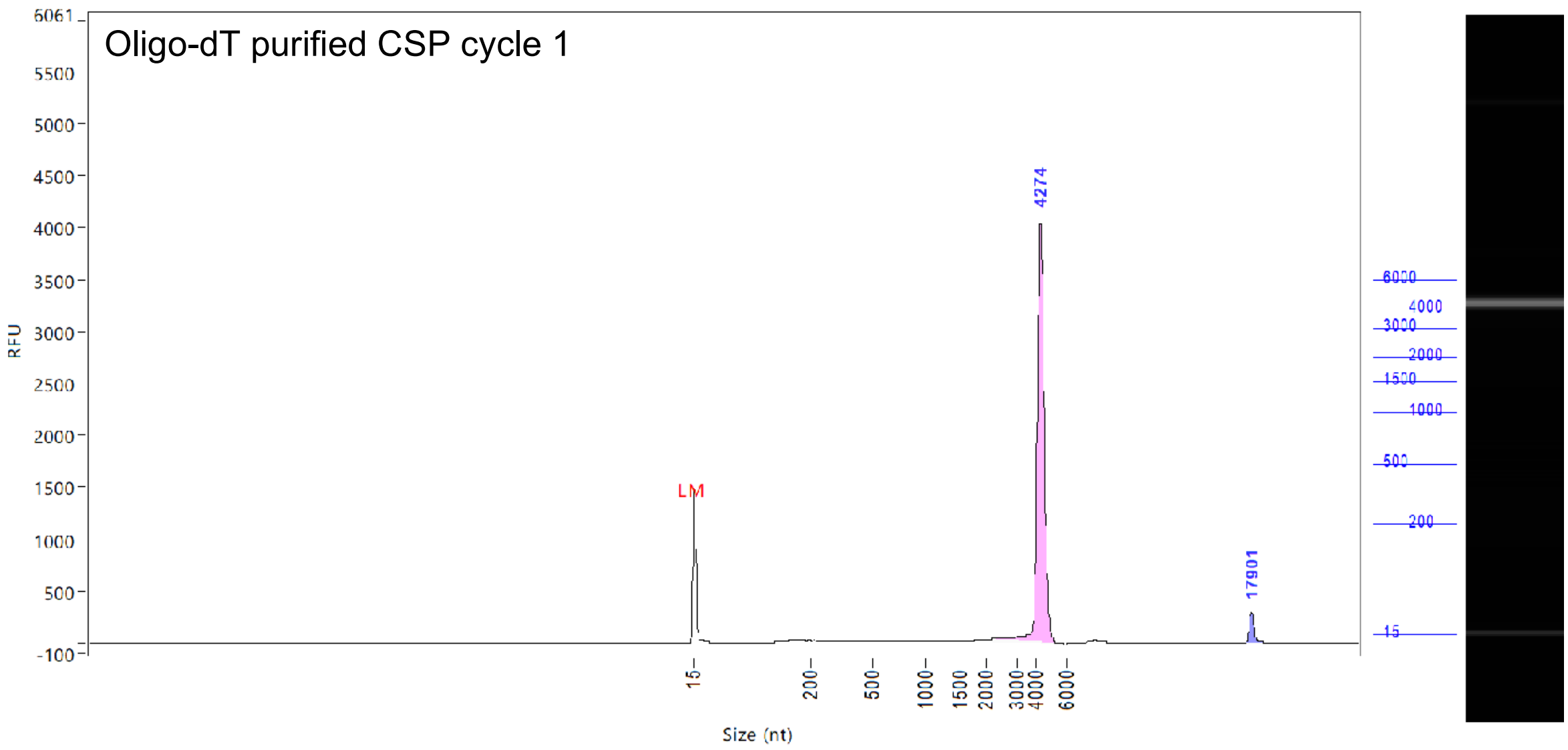

C

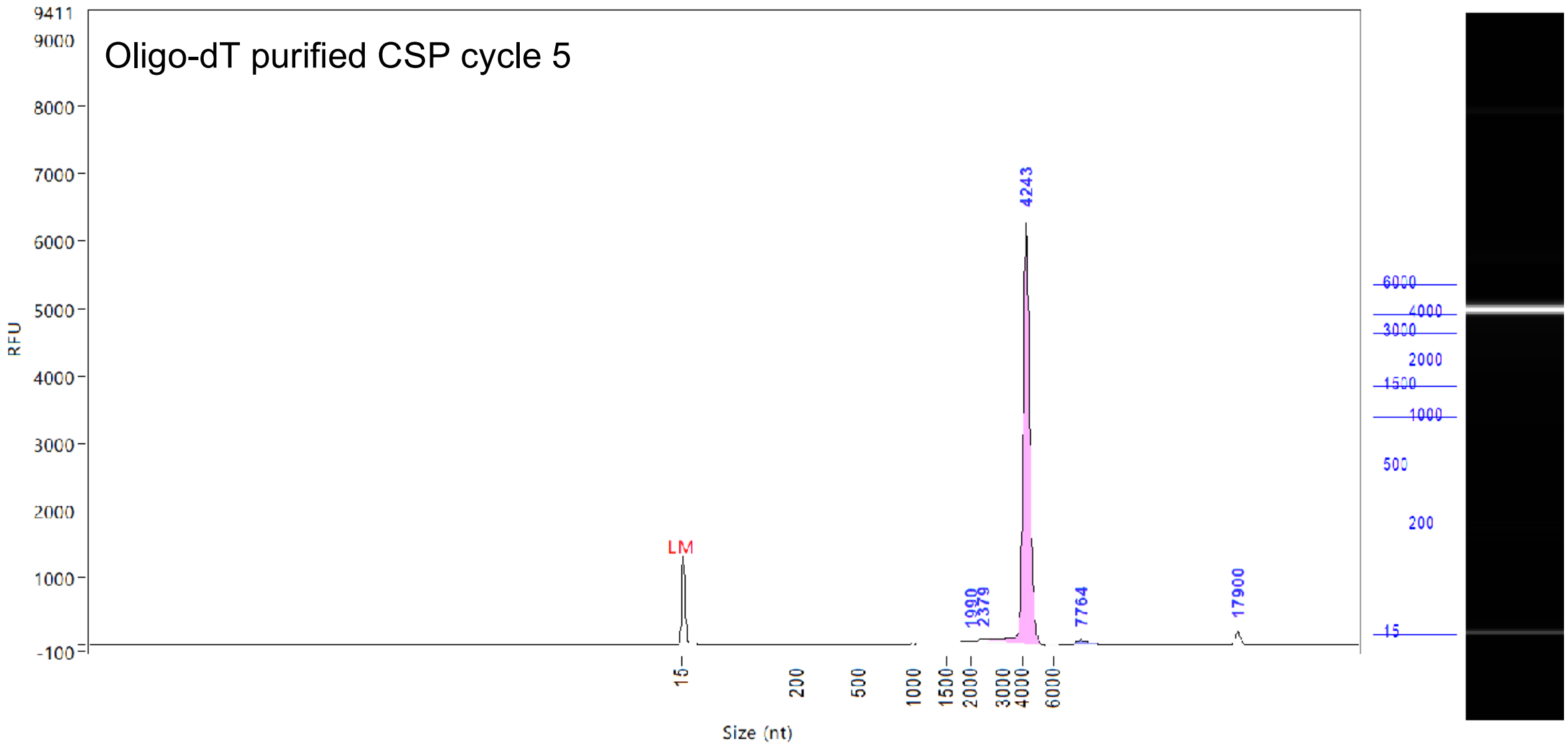

Figure S2. Feasibility of integrated IVT-oligo-dT recycling with a longer CSP transcript**. A,** Purified RNA yield per cycle and cumulative purified RNA output for SARS-CoV-2 Spike protein (CSP) mRNA produced using the CH_3COOK-based recycling workflow. Bars show purified RNA yield obtained in each cycle, with individual replicate values overlaid as dots; error bars indicate standard deviation. The black line with open circles represents cumulative purified RNA yield across successive cycles, referenced to the right y-axis. **B,C,** Representative electropherograms of purified CSP mRNA recovered after oligo-dT chromatography from cycle 1 and cycle 5, respectively. The comparable electropherogram profiles are consistent with preserved purified-product integrity across the recycling process. Crude CSP electropherograms were not available; therefore, these data were not used for crude-to-purified integrity comparison.

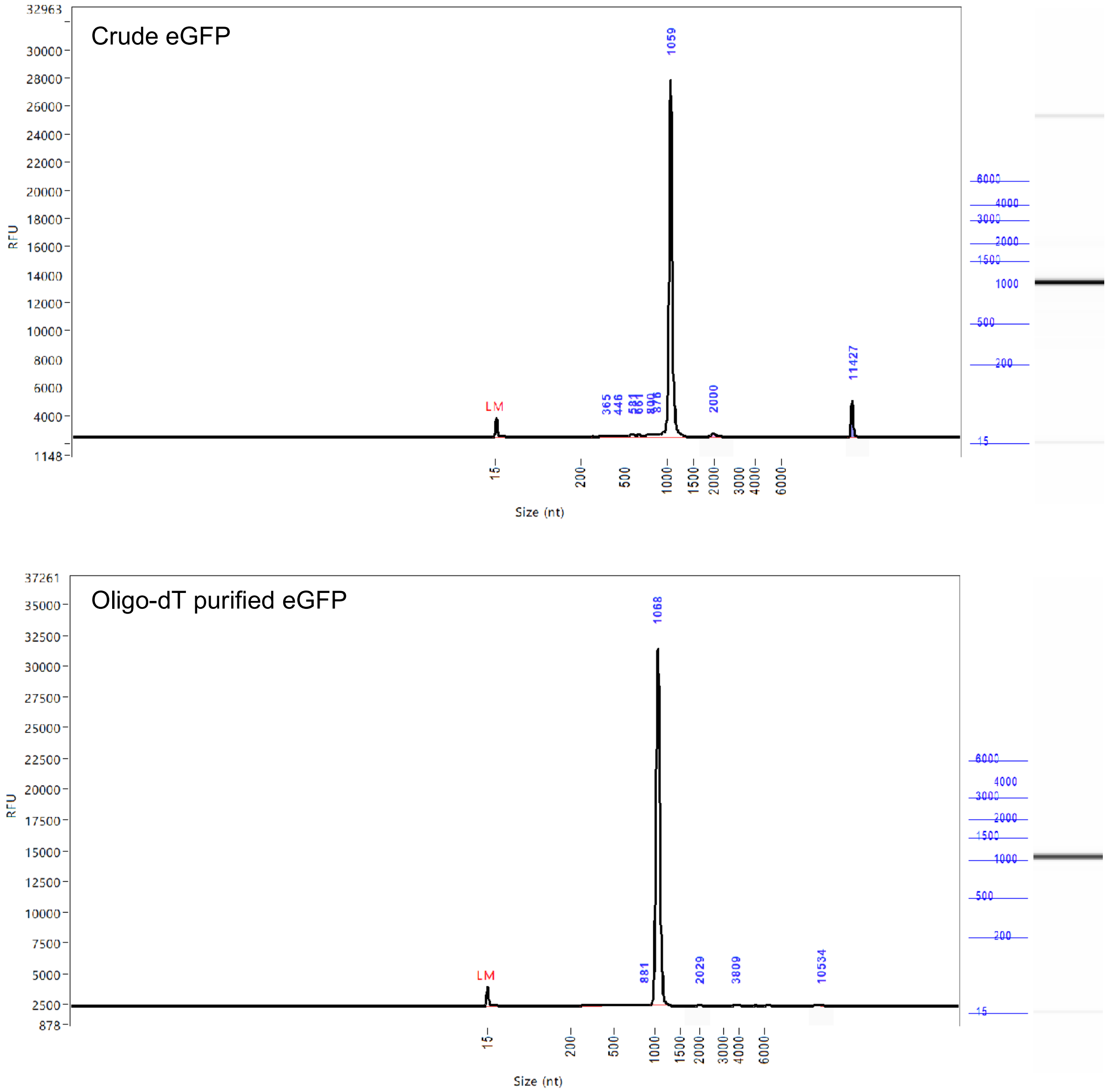

Figure S3. Representative electropherograms of crude and purified eGFP mRNA. Representative electropherograms are shown for crude eGFP mRNA after IVT and purified eGFP mRNA after oligo-dT chromatography.

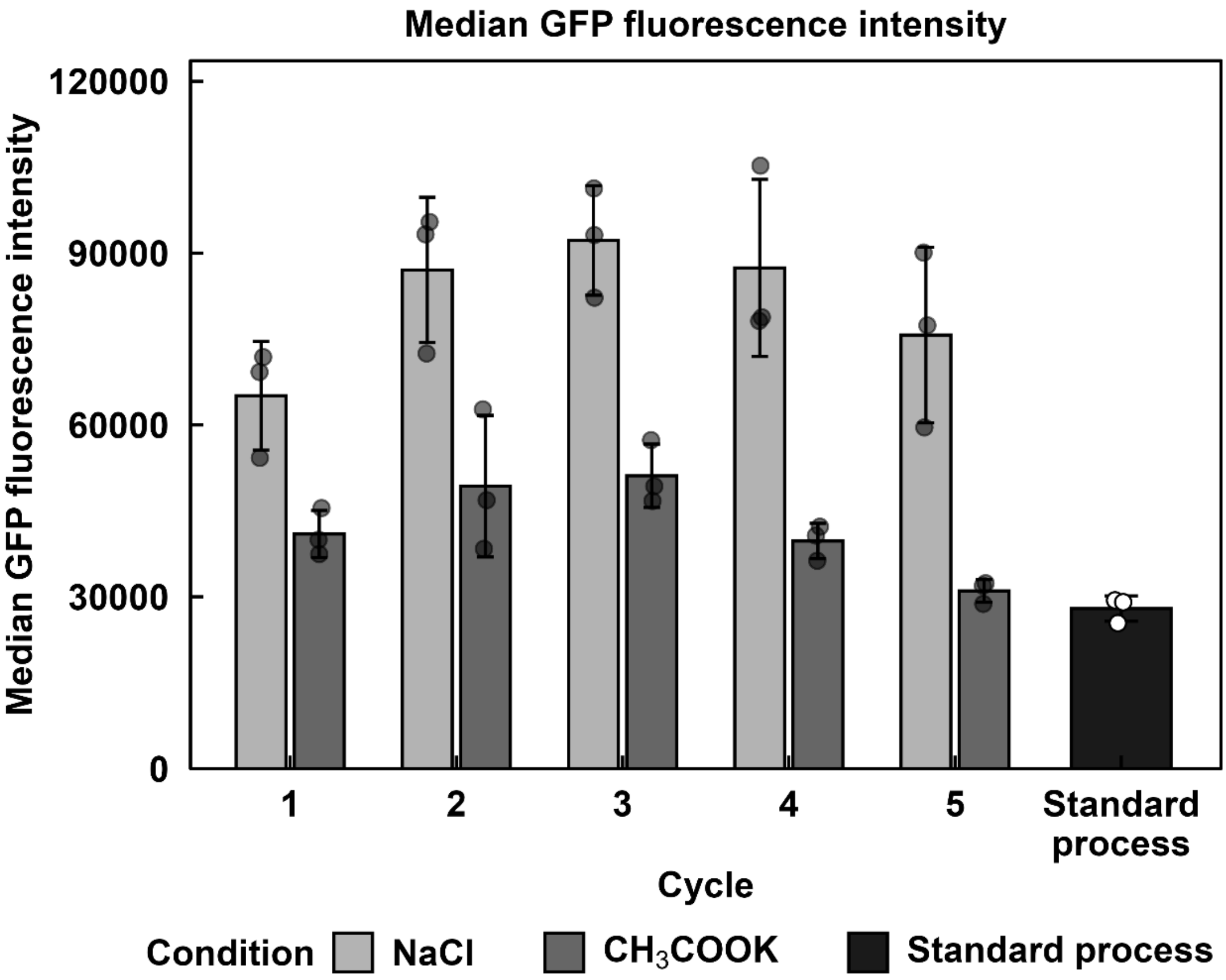

Figure S4. Median GFP fluorescence intensity following transfection with recycled mRNA**.** Median GFP fluorescence intensity (MFI) was measured in THP-1 cells transfected with eGFP mRNA generated using the standard batch IVT process or the integrated IVT-oligo-dT recycling workflow with NaCl or CH_3COOK buffers. Bars show mean values from three independent transfection experiments, with individual replicate values overlaid as points; error bars indicate SD. The data provide a complementary protein-expression readout to the percentage of eGFP-positive cells shown in **Figure 6A**.
